# Synthesis and biological activities of 3-aminoimidazo[1,2-α]pyridine compounds

**DOI:** 10.1186/s13065-025-01412-6

**Published:** 2025-02-22

**Authors:** Isra Al-Qadi, Michel Hanania, Ismail Warad, Nisreen Al-Hajj, Rand Hazzam, Yousef Salama, Saki Raheem, Nawaf Al-Maharik

**Affiliations:** 1 Department of Chemistry, Faculty of Science, An-Najah National University, Nablus, 00970 Palestine; 2 Department of Chemistry, Faculty of Applied Sciences, Technology and Engineering, Bethlehem University, Bethlehem, 00970 Palestine; 3 Department of Biomedical Sciences, Faculty of Medicine and Health Sciences, An-Najah National University, Nablus, 00970 Palestine; 4 School of Life Sciences, University of Westminster, 115 New Cavendish Street, London, W1W 6UW UK

**Keywords:** Anticancer, 3-aminoimidazole[1,2-α]pyridine, 4-chlorophenyl isonitrile, GBB-3CR, Multicomponent reaction

## Abstract

**Abstract:**

Despite their importance in cancer treatment, anticancer compounds face significant challenges due to drug resistance and low specificity, creating an urgent need for the discovery of more effective alternative. Herein, we report the synthesis of eleven 3-aminoimidazole[1,2-α]pyridine compounds **(9–19)** employing the one-pot Groebke-Blackburn-Bienayme three-component reaction (GBB-3CR). The cytotoxicity of the synthesised compounds was evaluated against three cancer cell lines (MCF-7, HT-29, B16F10) and a normal cell (MEF). Considering effectiveness and safety, the results demonstrated that among the eleven synthesised compounds, only compounds **12** and **14** exhibited high inhibitory activity against cancer cell lines. Compound **12** with a nitro group at the C-2 position and a *p*-chlorophenyl group at C-3 position, showed the highest inhibitory activity against HT-29, with an IC_50_ of 4.15 ± 2.93 µM. Additionally, compound **14**, with a tolyl moiety at the C-2 position and a *p-*chlorophenyl amine at C-3 position, can also be considered a promising bioactive product against B16F10, with an IC_50_ of 21.75 ± 0.81 µM. Further research on these compounds may yield more potent candidates for the development of new anticancer agents.

**Graphic abstract:**

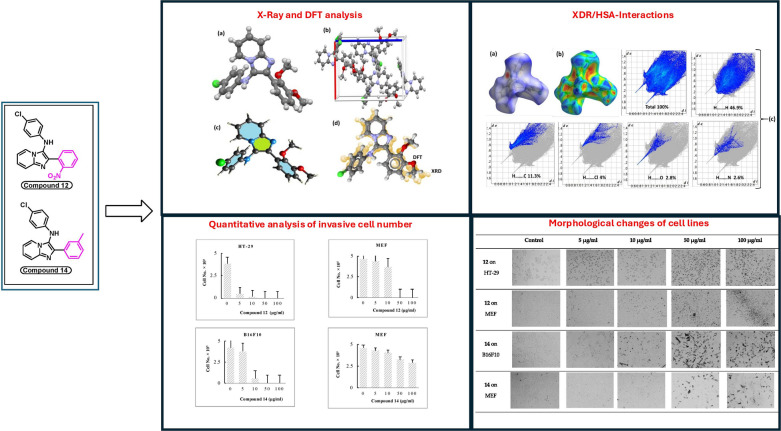

**Supplementary Information:**

The online version contains supplementary material available at 10.1186/s13065-025-01412-6.

## Introduction

**Fig. 1:**
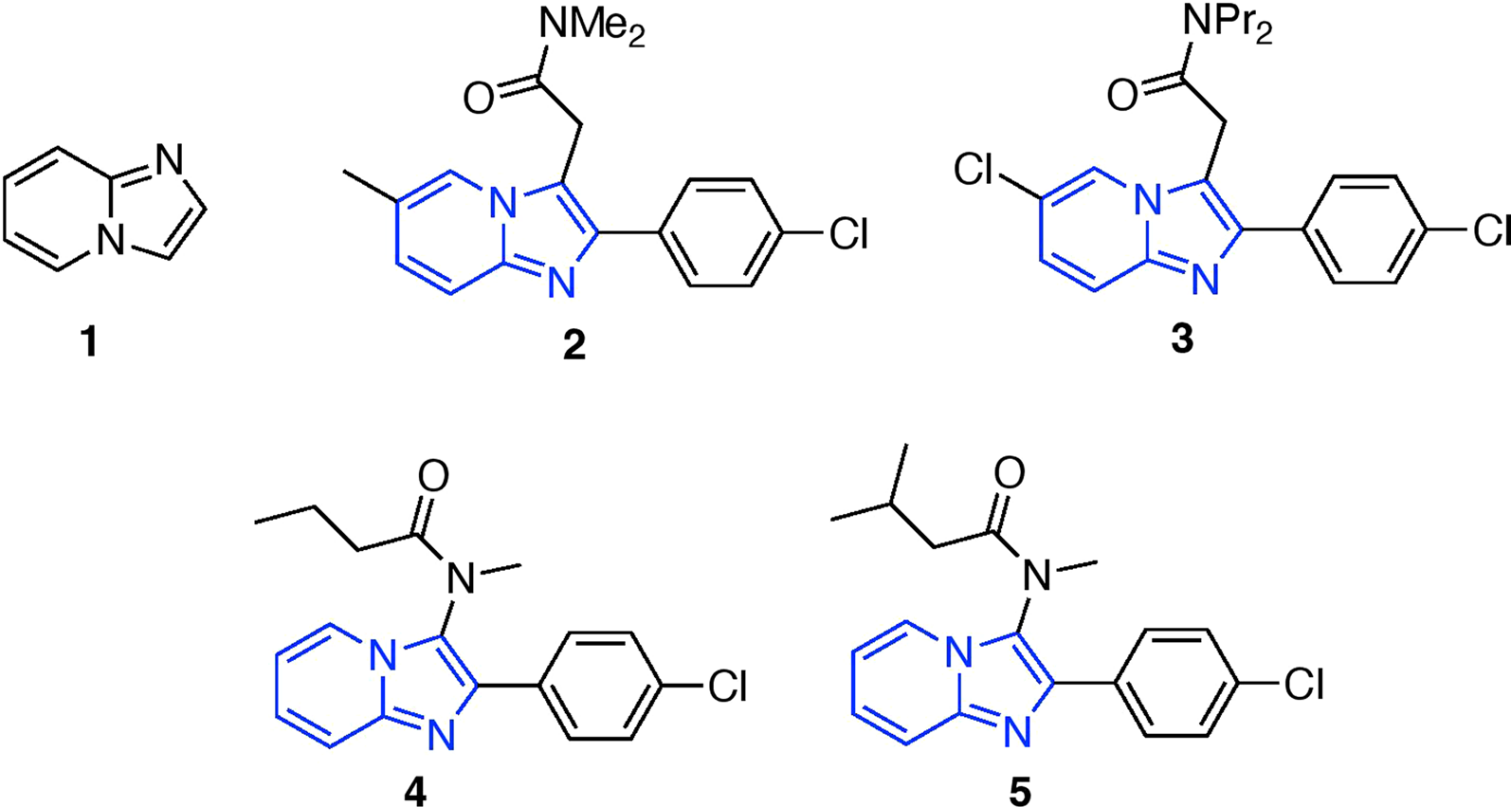
Chemical structure of imidazopyridine and derivatives featuring the imidazopyridine scaffold

Bioactive organic molecules often contain nitrogen-based heterocycles, which exhibit a broad spectrum of biological activities [1–10]. Imidazole[1,2-α]pyridines **1** play a crucial role as key structural constituents in several compounds. Recently, researchers have discovered that imidazole[1,2-α]pyridine scaffolds exhibit remarkable biological characteristics, serving various purposes such as antiviral [4, 11], antiprotozoal [12], anticancer [1–9, 13], and antimicrobial [14] activities. The various biological effects of these compounds have prompted continuous efforts to accelerate the development of novel synthetic methods for producing derivatives with structural variations at the 2- and 3- positions of the moiety. The synthetic methods encompass a range of techniques, including condensation reactions, oxidative coupling, one-pot multicomponent procedures, hydroamination reactions, and amino-oxygenation reactions [15]. The Groebke-Blackburn-Bienayme three-component reaction (GBB-3CR) is an efficient and cost-effective method for synthesizing imidazole[1,2-α]pyridines [16–18] offering the ability to modify the structure in three or more regions. The GBB-3CR has led to the development of various medicines and lead compounds, including zolpidem **2**, alpidem **3**, saripidem **4**, and necopidem **5** (Fig. 1). The GBB reaction involves one-pot acid-catalyzed condensation of aminoazines with aldehyde and isocyanides. Although there have been notable advancements in drug therapies over the last ten years, cancer remains a substantial health obstacle. Cancer treatment now includes advanced modalities beyond conventional methods. Natural product therapy uses bioactive compounds from plants to target cancer pathways with fewer side effects [19]. Exosome-base therapy utilizes exosomes for precise drug delivery to cancer cells [20]. Stem cell therapy offers potential in regenerating damages tissues or delivering targeted treatments to resistant tumors [21]. Gene therapy modifies faulty gene or actives the immune system to combat cancer [22]. Nanoparticle-based therapy enhances drug delivery and imaging, targeting cancer cells precisely while sparing healthy tissues [23, 24]. Additionally, radiation therapy and immunotherapy remain crucial, using high-energy radiation and boosting the immune system to effectively treat various cancers [25, 26]. These innovative approaches are revolutionizing cancer treatment with more effective and personalized options.

**Fig. 2:**
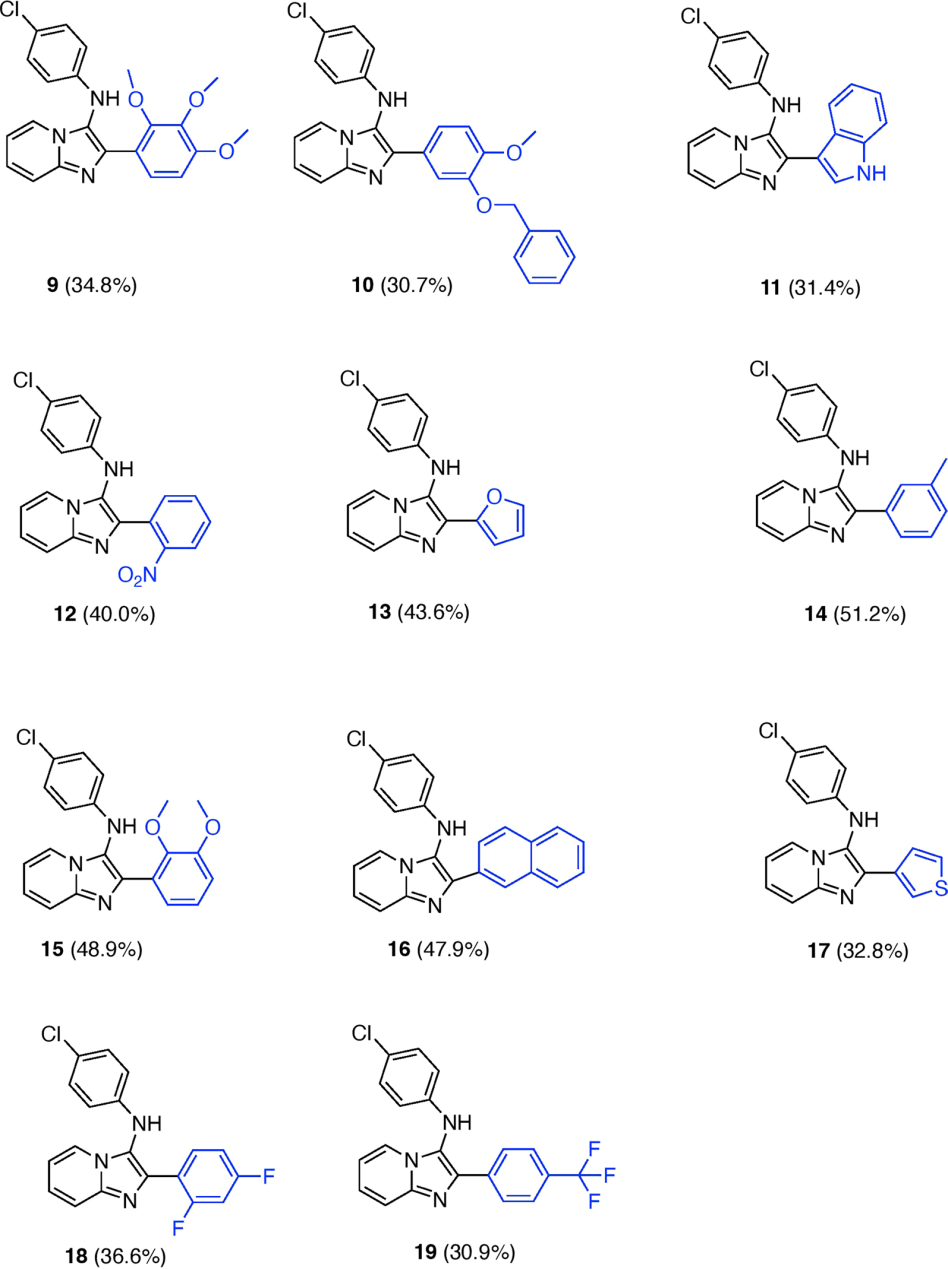
Structure–activity relationships of the synthesized compounds 9–19

This study aims to synthesize imidazo[1,2-α]pyridine derivatives (Fig. 2) utilizing GBB-3CR and to assess their potential as anticancer agents. The structural determination of the novel compounds was accomplished using ^1^H- and ^13^C-NMR, IR, and XDR-single techniques.

## Results and discussion

### Synthesis

**Fig. 3:**
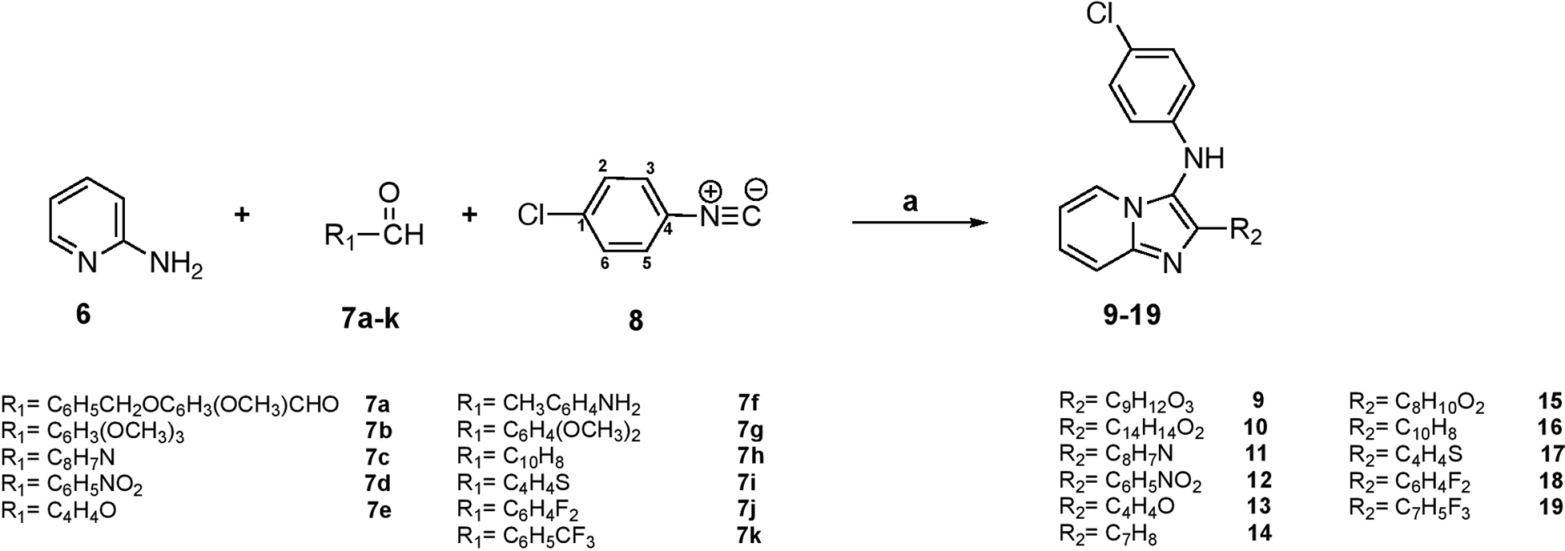
Reagents and conditions: **a** p -TsA.H 2 O or Sc(OTf) 3 , MeOH: CH 2 CI 2 (1:1) or MeOH only., 1 h, 50 °C

The imidazo[1,2-α]pyridine derivatives were produced using acid-catalyzed GBB-3CR, as illustrated in Fig. 3. The procedure involved the condensation of 2-aminopyridine **6** with various aldehydes **7a-k**, employing a small quantity of *p*-toluenesulfonic acid or scandium triflate as catalysts [18]. This was followed by cyclization of the resulting imide intermediate with 4-chlorophenyl isocyanide **8**. The presence of electron-donating (ED) groups on the benzaldehydes, such as the methoxy group in **15**, the methyl group in **14** and the benzene rings in **16**, may result in moderate yields, higher than the yield obtained for compound **19**, which bears the electron-withdrawing (EW) trifluoromethyl (CF_3_) group (Fig. 2). The product’s moderate yield (30–50%) could be ascribed to the utilization of repeated flash chromatography and recrystallization to obtain the very pure compounds required for biological screening. The compounds’ purity was evaluated by HPLC, HRLM and elemental analysis. Additionally, the structure of the key pharmacophore, imidazo[1,2-α]pyridine, was validated using single-crystal X-ray crystallography on compound **15**.

### X-Ray and DFT analysis

**Fig. 4:**
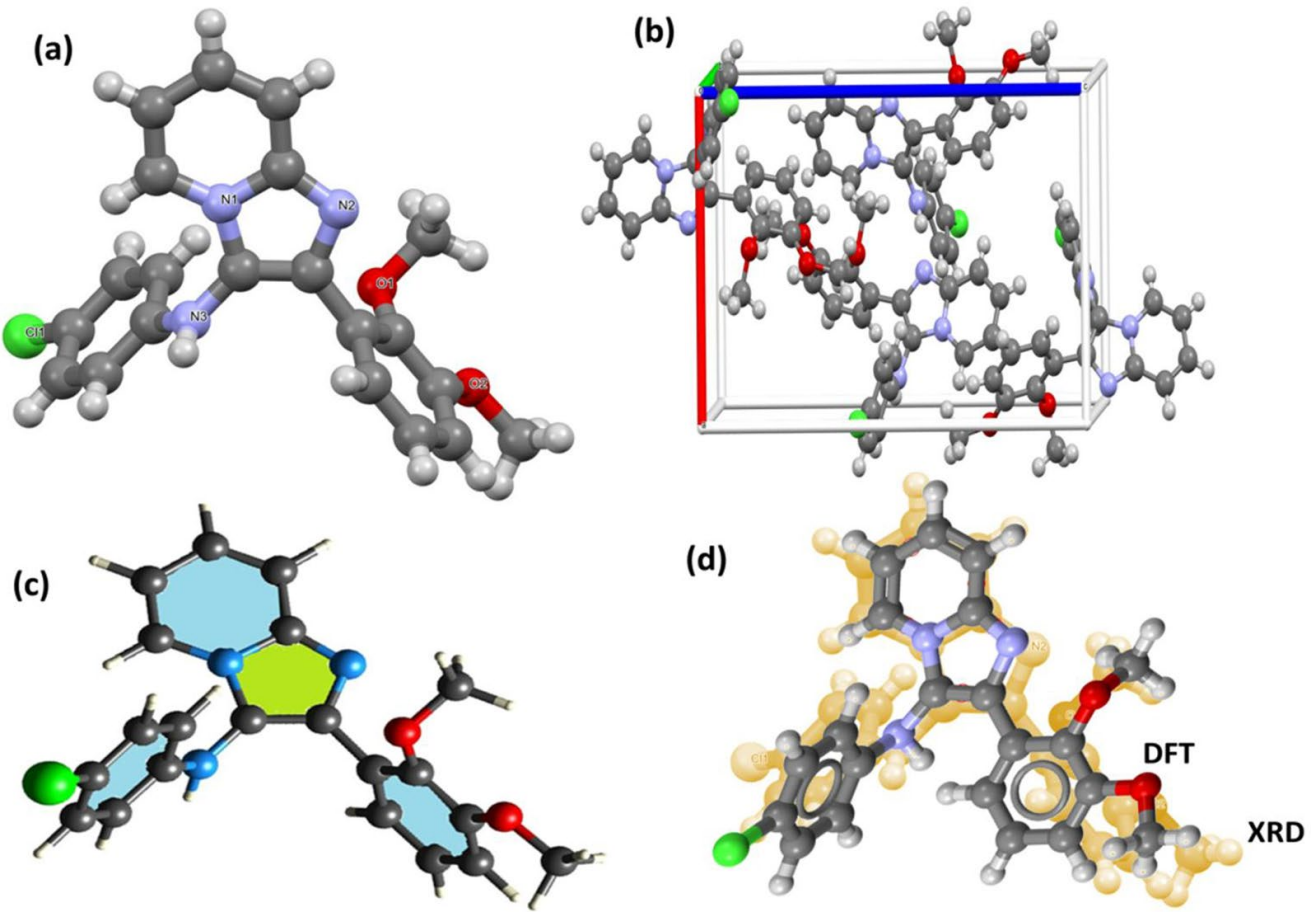
**a** 3D-XRD, **b** B3LYP/molecular struccture, **c** DFT/XDR structure matching and **d** crystal packing module

Figure 4 shows the X-ray diffraction (XRD) and density functional theory (DFT) analyses conducted to verify the three-dimensional arrangement of compound **15**. This compound was used as a reference because all the compounds synthesised in this study feature three aromatic rings with an imidazole[1,2-α]pyridine unit. Table S1 and Fig. 4a depict the XRD-ORTEP, while Fig. 4b highlights the DFT-optimized molecular structure. Compound **15** was crystallized in an orthorhombic crystal system with the P2_1_2_1_2_1_ space group. The crystal structure obtained has the following lattice parameters: a = 10.2676 Å, b = 11.1678 Å, and c = 16.8317 Å. X-ray measurements showed the presence of three aromatic rings with the imidazo[1,2-α]pyridine unit in a semi-perpendicular shape to each other, forming a fun wing-like structure. The formation of the *N*-phenylimidazo unit has also been confirmed, as the N-H unit was identified by SC-XRD, as shown in Fig. 4a. A densely orthorhombic molecular packing structure with a Z-factor of 4 has been observed in the crystal lattice of the desired molecule (Fig. 4b). The DFT optimization structure (Fig. 4c) confirmed the SC-XRD collections regarding the 3-D structure, atom orientation, and structural parameters like angles, torsions, and bond lengths, as seen in Fig. 4d and Table S1.

### XDR/HSA-Interactions

**Fig. 5:**
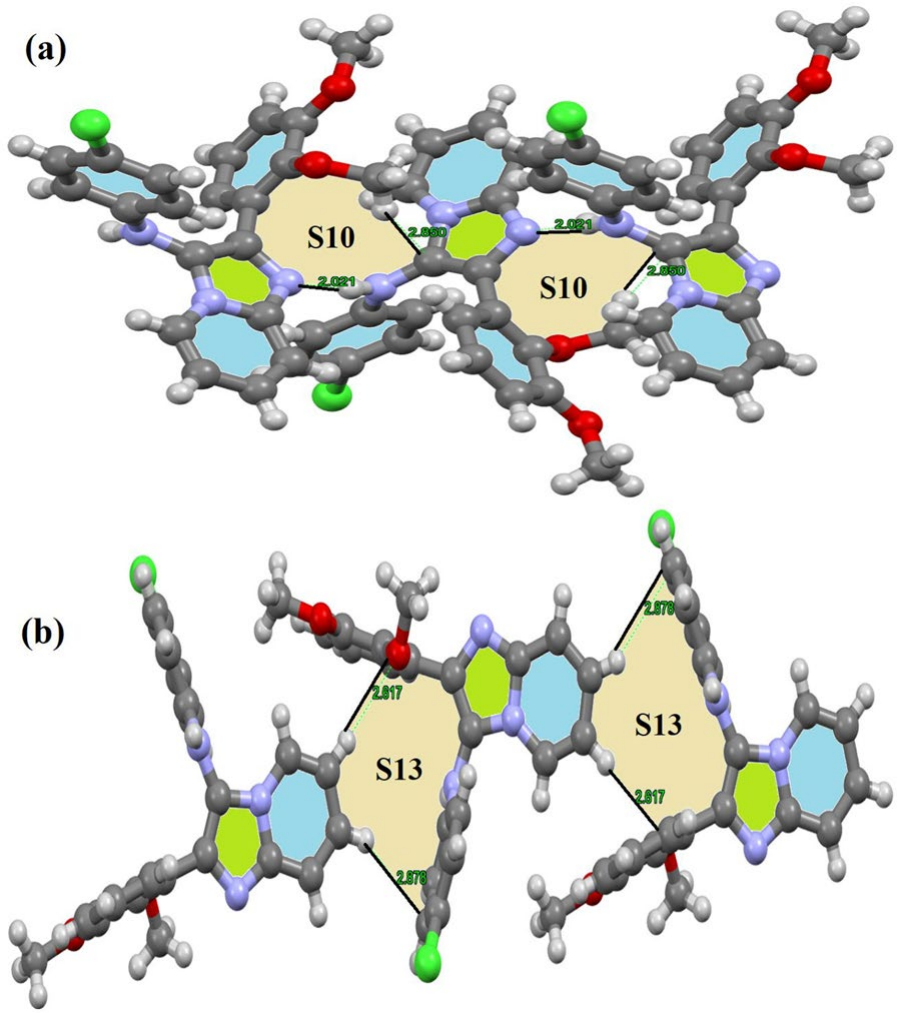
**a** S10, and **b** S13 synthons formed in the X-ligand lattice

The synthesized compound **15** had a molecular structure that was defined by the formation of many non-classical and classical H-bond interactions, such as C-H…O/C-H…Cl and N-H…N. These interactions lead to the creation of two main types of new 2D-synthons without 1D-chain interactions. The molecular structure of the desired ligand is distinguished by the accumulation of classical N-H…N H-bond and C-H…C = N interactions with 2.021 and 2.850 Å bond lengths, respectively, constructing two 2D-S10 synthons per molecule (Fig. 5a). The second synthon was S13 (2 per molecule), formed by combining C-H…O and C-H…πPh interactions with 2.617 and 2.878 Å bond lengths, respectively (Fig. 5b).

**Fig. 6:**
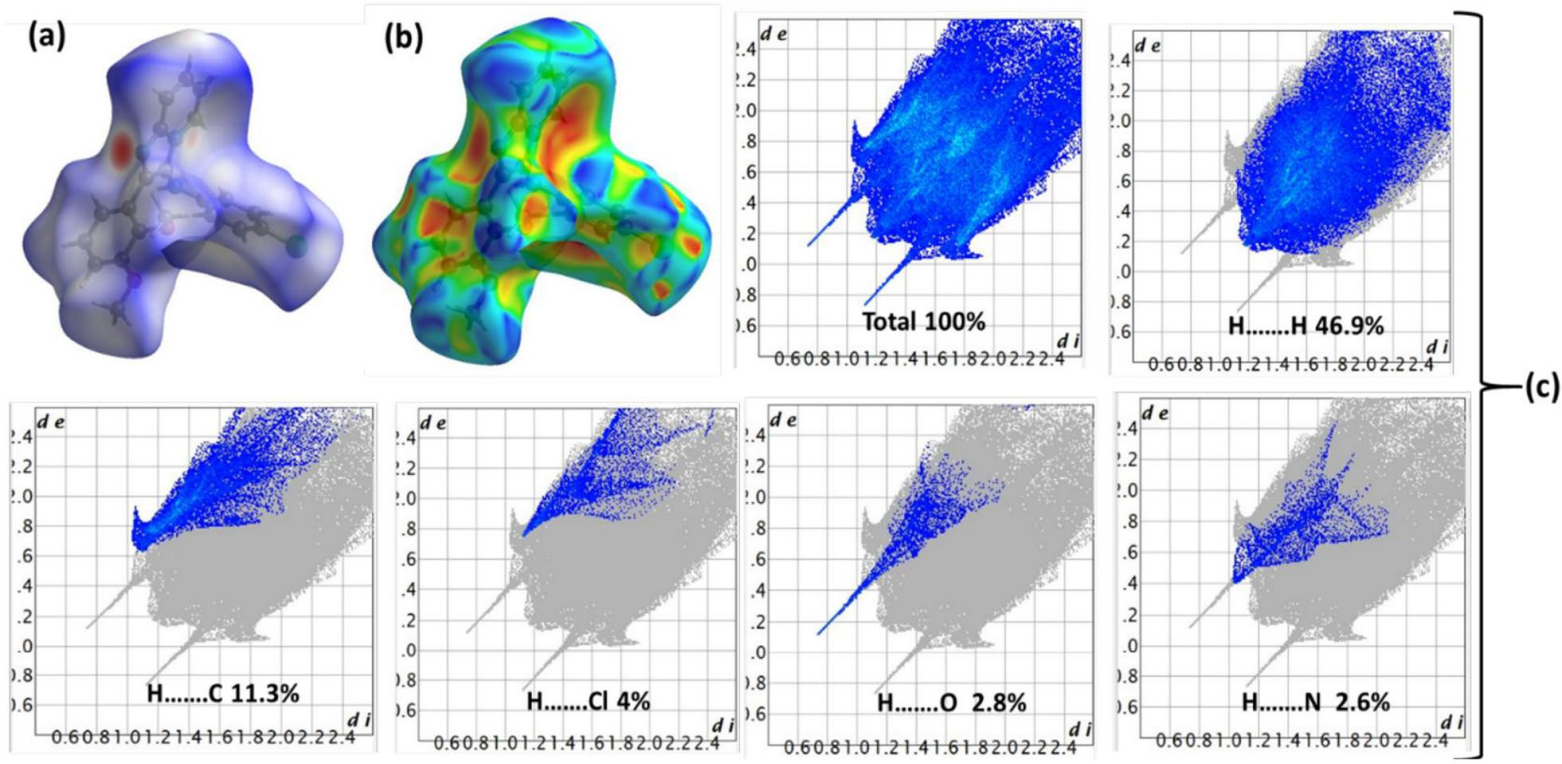
**a** dnorm **b** shape-index, and **c** 2D-FP atom-to-atom ration

Figure 6 presents the HSA analysis, which provides vital information about the intermolecular interactions taking place within the computed molecule and its surroundings. The HSA analysis utilizes a color-mapped representation that ranges from − 0.462 (shown as blue) to 1.567 (represented as red). The dnorm surface (Fig. 6a) displays five prominent red areas, indicating the type and strength of interactions per molecule. Moreover, the dnorm surface shows significant interactions, such as two classical short N–H…N H-bond interactions per molecule and several non-classical C–H…O H-bond interactions. Furthermore, the dnorm surface exhibits the major interactions required, including the classical presence of two short N–H…N hydrogen bond interactions per molecule, as well as multiple non-classical C–H…O hydrogen bond interactions.

The shape index (Fig. 6b) indicated the presence of electron concentrations surrounding functional groups such as C–O, C–Cl, and C–N, which were visually represented by surfaces in red, blue, and orange. The presence of blue surrounding surface atoms, particularly hydrogen atoms, along with the red color representing O, N, Cl, and other highly electronegative atoms (Fig. 6c), promotes the formation of polarized bonds, such as hydrogen bonds. In addition, the 2D-FP computations depicted in Fig. 6c, verified the presence of intermolecular contacts and determined the proportion of atom-to-atom interactions (H^…^.X%) in the following order: H….H(46.9%) > C….H(11.3%) > Cl….H(4%) > O….H (2.8%) > N….H(2.6%). The contributions of the H-atoms were found to be greater than those of the C-atoms, which were in turn greater than those of the Cl-atoms (Fig. 6c); this was confirmed by practical XRD observations in the crystal lattice.

## Biological activity

### Anticancer activity

The cytotoxicity of the synthesized imidazo[1,2-*a*]pyridine compounds **(9–19)** were screened against breast (MCF-7), colon (HT-29) and melanoma (B16F10) cancer cell lines, as well as normal cell line (MEF) as shown in Table 1.

**Table 1:** In vitro antiproliferative activity data of compounds 9–19

Cpd’s designation	^a^IC_50_ (µM)			
	MCF-7	HT-29	B16F10	MEF
Paclitaxel (Taxol)	0.00834	0.0166	55.7	17.23
	> 200	> 200	> 200	> 200
	> 200	> 200	> 200	18.5 *±* 0.33
	20.47 *±* 0.10	18.34 *±* 1.22	39.2 *±* 1.84	1.84 *±* 0.83
	30.88 *±* 14.44	4.15 *±* 2.93	64.81 *±* 15.78	40.54 *±* 4.34
	66.48 *±* 37.87	48.31 *±* 0.53	197.06 *±* 14.42	32.93 *±* 0.09
	195.26 *±* 19.89	44.45 *±* 0.15	21.75 *±* 0.81	> 200
	> 200	> 200	> 200	> 200
	84.05 *±* 3.37	93.08 *±* 0.61	> 200	> 200
	> 200	> 200	> 200	> 200
	14.81 + 0.20	10.11 *±* 0.70	14.39 *±* 0.04	16.31 *±* 0.08
	> 200	85.50 *±* 18.83	195.45 *±* 15.30	63.85 *±* 2.33

^a^ The IC_50_ values (µM) represent an average of two independent experiments (mean ± SD)

Cytotoxic analysis of synthesized compounds revealed that imidazopyridine compound **18** showed the highest cytotoxicity against MCF-7 with IC_50_ = 14.81 *±* 0.20 µM, followed by compound **11**, which has an indole moiety at the C-2 position, with IC_50_ = 20.47 *±* 0.10 µM. However, compound **12** with an EW group (nitro) is effective against MCF-7 with IC_50_ = 30.88 *±* 14.44 µM and had moderate cytotoxicity against B16F10 (IC_50_ = 64.81 *±* 15.78 µM). Compound **13**, bearing a furan moiety at the C-2 position, showed weak bioactivity against B16F10 (IC_50_ = 197.06 *±* 14.42 µM) and moderate anticancer activity against MCF-7 (IC_50_ = 66.48 *±* 37.87 µM). On the other hand, compounds **9**, **10**, **15** and **17** were ineffective against MCF-7 as well as the other two cell lines.

The imidazopyridine compound **12**, with a nitro group on the moiety substituted at the C-2 position and a *p*-chlorophenyl ring at the C-3 position, demonstrated the highest inhibitory activity against HT-29 cell, with an IC_50_ value of 4.15 *±* 2.93 µM. The next highest level of cytotoxicity against this cancer cell line was exhibited by compound **18**, with an IC_50_ value of 10.11 *±* 0.70 µM. Additionally, compound **11** also showed high anticancer activity against this cancer cell line with IC_50_ value of 18.34 *±* 1.22 µM. This value was similar to the high activity of this compound against MCF-7, in contrast to its moderate cytotoxicity against B16F10 (IC_50_ = 39.20 *±* 1.84 µM). Compounds **13** and **14** also exhibited moderate inhibitory activity against HT-29, with IC_50_ values of 48.31 *±* 0.53 and 44.45 *±* 0.15 µM, respectively.

The imidazopyridine compound **18**, with a 2,4 difluorophenyl moiety substituted at the C-2 position and a *p*-chlorophenyl amine at the C-3 position, displayed the highest inhibitory activity against B16F10 with an IC_50_ of 14.39 *±* 0.04 µM. Compound **14**, which had a tolyl moiety at the C-2 position and *p*-chlorophenyl amine at the C-3 position, showed the next highest cytotoxicity value, with an IC_50_ of 21.75 *±* 0.81 µM. However, compound **16**, which contains a naphthalene moiety at the C-2 position, exhibited good anticancer activity, with an IC_50_ values of 84.05 *±* 3.37 and 93.08 *±* 0.61 µM against MCF-7 and HT-29, respectively, while it was inactive against B16F10. Additionally, compound **19**, which had an EW group (trifluoromethyl) at the C-2 position, was ineffective against MCF-7 and B16F10 (IC_50_ *≥* 200 µM); however, it showed good inhibitory activity against HT-29 with an IC_50_ of 85.50 *±* 18.83 µM.

**Fig. 7:**
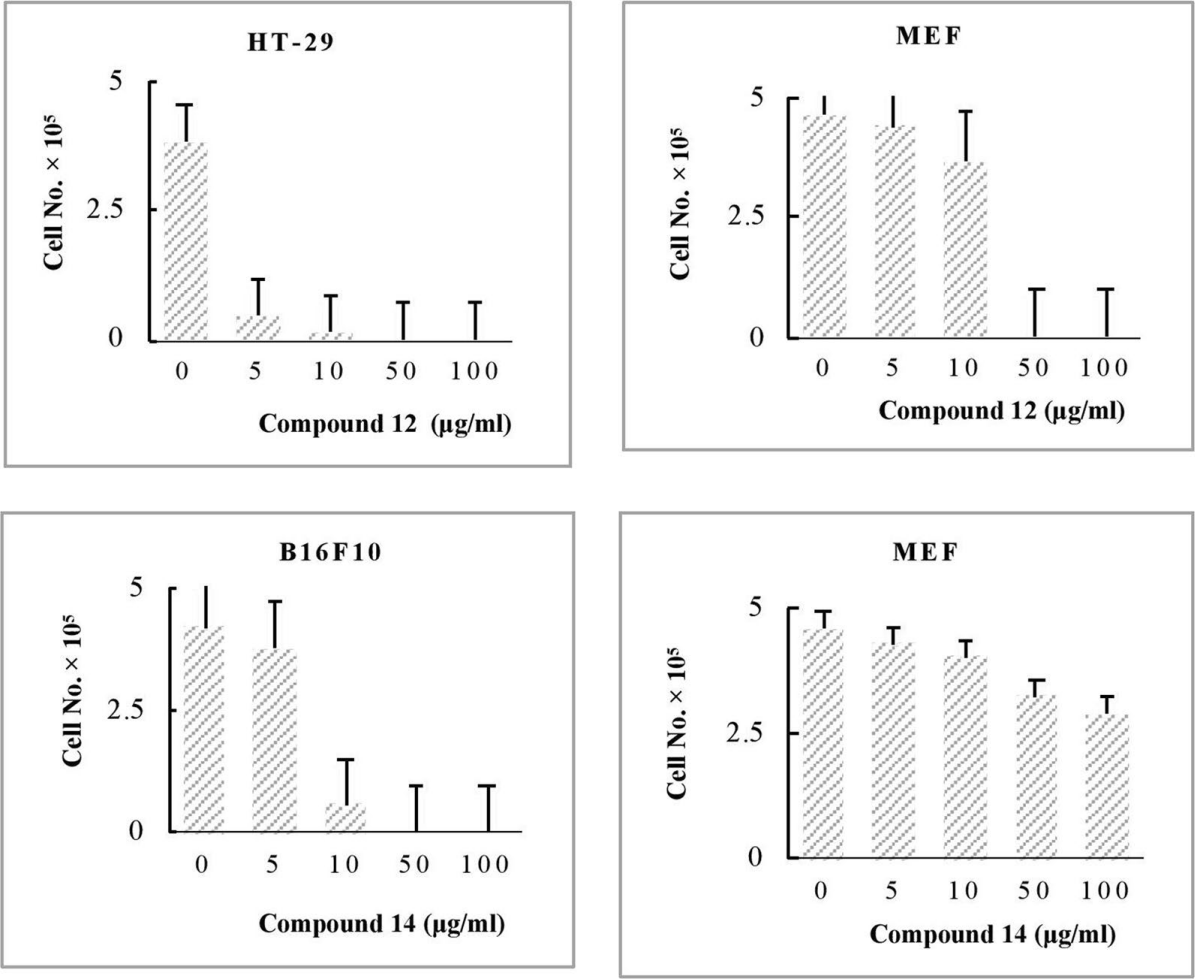
Quantitative analysis of invasive cell number

Therefore, this study conducted a further assessment of the cytotoxic effects of the synthesized compounds on the normal cell line MEF. Among the promising bioactive compounds, compound **11** was the most cytotoxic against normal cells (IC_50_ = 1.84 ± 0.83 µM), followed by compounds **18** (IC_50_ = 16.31 ± 0.08 µM) and 13 (IC_50_ = 32.93 ± 0.09 µM). Other bioactive compounds that showed moderate cytotoxicity against MEF include compound **12** (IC_50_ = 40.54 ± 4.34 µM) and compound **19** (IC_50_ = 63.85 ± 2.33 µM). As previously described, compound **11** showed high toxicity against MCF-7 and HT-29 (IC_50_ < 20.47 µM), but it was also very toxic against normal cells (IC_50_ < 1.84 µM). Additionally, compound **13** showed cytotoxicity against normal cells with an IC_50_ of 32.93 ± 0.09 µM, which is lower than the IC_50_ reported against cancer cell lines (IC_50_ > 45.00 µM). Although compound **18** was potent against the three cancer cell lines (IC_50_ < 15.00 µM), it was also cytotoxic against MEF, with a value approximately similar (IC_50_ = 16.31 ± 0.08 µM). For these reasons, the bioactivity of compounds **11**, **13**, and **18** was not considered at this stage. Compound **12** is a more potent against HT-29, with an IC_50_ of 4.15 ± 2.93 µM, while its cytotoxicity against normal cells is tenfold lower (IC_50_ = 40.54 ± 4.34 µM). For the same reasons, compound **14** can be considered a promising bioactive product against B16F10, with an IC_50_ of 21.75 ± 0.81 µM versus > 200 µM against MEF. These IC_50_ values demonstrate the high selectivity of the two promising compounds **12** and **14**, against cancer cells compared to normal cells, as confirmed by the histograms in Fig. 7.

**Fig. 8:**
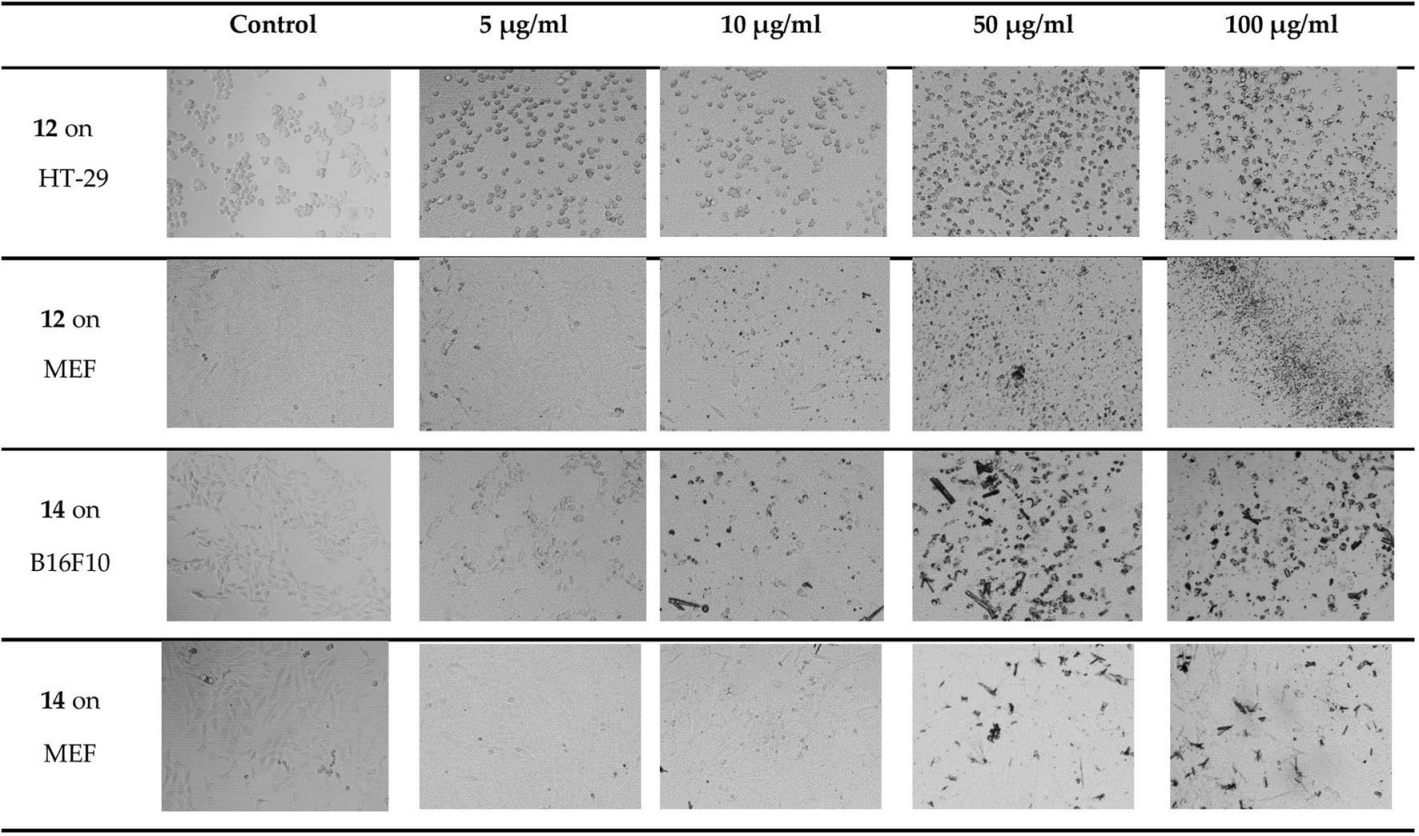
Morphological changes of the most potent synthesized compounds treated with cancer and normal cell lines and controls (untreated cancer cells)

In comparison to the control (untreated cancer cells), the three cancer cell lines (MCF-7, HT-29, and B16F10) were investigated for morphological changes after being treated with the synthesized compounds 9–19. As shown in Fig. 8, cells of the HT-29 and B16F10 cell lines showed shrinkage and lost their original form when treated with compounds 12 and 14 at high concentrations (*≥* 50 µg/ml).

## Conclusions

The present study describes the synthesis of imidazo[1,2-a]pyridine compounds (9–19) utilizing an acid-catalyzed GBB-3CR. All synthesized compounds were screened for cytotoxic activity against the cancer cell lines MCF-7, HT-29, and B16F10, as well as the normal MEF cell line, using the Trypan Blue Exclusion Assay. Analysis of the synthesized compounds, based on their efficacy and safety, indicated that imidazopyridines 12 and 14, with para-chlorophenyl at C-3, and nitro and methyl groups substituted on the phenyl ring at C-2, respectively, were potent anticancer agents against the HT-29 (colon) and B16F10 (melanoma) cancer cell lines (Fig. 8).

While this study synthesized eleven imidazo[1,2-a] analogues with variations exclusively in the aldehyde component, the choice of p-chlorophenyl isonitrile was primarily due to its known reactivity and effectiveness in facilitating the desired reaction under specified conditions. Additionally, it provided a consistent structural framework for assessing the impact of aldehyde variations on the biological activity of the resulting compounds. One constraint of this study was the focus on a single isocyanide, which limited the diversity of structural analogues. Future studies will aim to explore a broader range of isocyanides and various 2-aminoazines to evaluate their influence on reaction scope and the biological properties of the synthesized analogues.

## Experimental

### General information

The chemical compounds and solvents, procured from Sigma-Aldrich, were utilized without undergoing additional purification procedures. The 4-chlorophenyl isocyanide was synthesized using the methodology outlined by Weber and Gokel (1972) [27]. HPLC-grade methanol (MeOH) was acquired from Sigma-Aldrich and utilized for synthesis processes conducted under inert gas (N_2_) conditions. ^1^H-, ^19^F-, and ^13^C-NMR were recorded in CDCl_3_ on 500 MHz Bruker AV III spectrometer. Chemical shifts are represented by the *δ* value (ppm), whereas coupling constants (*J*) are expressed in Hertz (Hz). The PerkinElmer 2400 analyzer was used to obtain the elementary analysis of C, H, and N. High-resolution mass spectra data (HRMS) were collected using the LTQ-Orbitrap XL (Thermo Fisher). FT-IR spectra were recorded on a Thermo Scientific Nicolet 1s5-Id3 Fourier transform infrared spectrophotometer. The progress of the reaction was monitored using aluminium-supported Thin Layer Chromatography (TLC) silica gel sheets (DC-Fertigfolien ALUGRAM® SIL G/UV254), and the spots were visualized using UV fluorescence. Moreover, silica gel from Sigma-Aldrich with a 60 Å pore size, 230–400 mesh, and 40–63 μm particle size under 5 psi compressed air was utilized for flash column chromatography. Solvents were evaporated using a Buchner Rotary Evaporator. Melting points were measured using Electrothermal Digital Mel-Temp 3.0 Melting Point apparatus. HPLC Waters Alliance e2695 equipped with a 2998 PDA detector (Waters Corporation, MA, USA) was used for the analysis of the synthesized compounds. RP C18 column (Restek Roc, 150 × 4.6 mm, 3 μm) was used, with a flow rate of 0.8 ml/minute. The DFT calculations were performed using the Gaussian09W software, employing the DFT/B3LYP/6-311G (d, p) methodology [28].

#### N-(4-chlorophenyl)−2-(2,3,4-trimethoxyphenyl)imidazo[1,2-a]pyridin-3-amine (9)

A mixture of 2-aminopyridine (282 mg, 3.0 mmol) and 2,3,4-trimethoxybenzaldehyde (589 mg, 3.0 mmol) containing Sc(OTf)_3_ (74 mg, 0.15 mmol) and anhydrous Na_2_SO_4_ (300 mg) in MeOH: dichloromethane (1:1, 15 mL) was stirred under a nitrogen atmosphere for one hour at 50 ^o^C. A solution of 4-chlorophenyl isocyanide (413 mg, 3.0 mmol) in MeOH (3 mL) was then added. After stirring at 60 ^o^C for 3 h, the mixture was cooled to room temperature and stirred overnight. Water (10 mL) was added to the reaction mixture and extracted with ethyl acetate (3 × 15 mL). The combined organic layers were washed with water (20 mL), dried over Na_2_SO_4_, filtered, and the solvent was evaporated under reduced pressure. The resultant residue was recrystallized twice with 95% ethanol to provide the pure compound **9** (429 mg, 34.8%) (Figure S1). MP: 219–219.8 °C; IR (cm^−1^): 3221(N–H str.), 2946 (C–H str. in CH_3_), 1598 (C = N str.), 1575 (C = C str.), 1489 (aromatic C = C str.), 1463 (C–H bending in CH_3_), 1286 (C–O str.), 821 (C–Cl str.) (Figure S2); ^1^H NMR (400 MHz, CDCl_3_) δ ppm: 7.72 (dt, *J* = 6.8, 1.2 Hz, 1H), 7.66 (dt, *J* = 9.1, 1.1 Hz, 1H), 7.52 (d, *J* = 8.7 Hz, 1H), 7.22 (ddd, *J* = 9.1, 6.7, 1.3 Hz, 1H), 7.07 (d, *J* = 8.8 Hz, 2 H), 6.81 (dt, *J* = 8.8, 1.2 Hz, 2 H), 6.77 (s, 1H), 6.34 (d, *J* = 8.9 Hz, 2 H), 3.93 (s, 3 H), 3.89 (s, 3 H), 3.72 (s, 3 H) (Figure S3); ^13^C NMR (101 MHz, CDCl_3_) δ ppm: 153.8, 150.9, 143.3, 142.2, 129.3, 125.5, 124.5, 124.2, 123.1, 120.7, 117.7, 115.5, 112.0, 108.6, 62.1, 61.2, 56.1. Anal. Calcd for C_22_H_20_CIN_3_O_3_ (409.87): C, 64.47; H, 4.92; N, 10.25%, found: C, 64.41; H, 8.64; N, 10.27%; HRMS *m/z* calcd for C_22_H_19_CIN_3_O_3_ ([M–H]^-^) 408.1193, found 408.1114 (Figure S4).

#### 2-(3-(Benzyloxy)−4-methoxyphenyl)-N-(4-chlorophenyl)imidazo[1,2-a]pyridin-3-amine (10)

A mixture of 2-aminopyridine (282 mg, 3.0 mmol) and 3-benzeloxy-4-methoxy-benzaldehyde (727 mg, 3.0 mmol) containing Sc(OTf)_3_ (74 mg, 0.15 mmol) and anhydrous Na_2_SO_4_ (300 mg) in MeOH: dichloromethane (1:1, 15 ml) was stirred under a nitrogen atmosphere for one hour at 50 ^o^C. The mixture was then subjected to the addition of a solution of 4-chlorophenyl isocyanide (413 mg, 3.0 mmol) in MeOH (3 mL), followed by the procedure described for **9**. The resultant residue was purified by flash chromatography (SiO_2_, dichloromethane/ethyl acetate in 6:4 ratio) to yield the desired compound **10** (419 mg, 30.7%) (Figure S5). MP: 205.8–207.3 °C; IR (cm^−1^): 3133 (N-H str.), 2931 (C–H str. in CH_3_), 1585 (C = C str.), 1490 (aromatic C = C str.), 1467 (C–H bending in CH_2_), 1257 (C–N str.), 1138 (C-O str.), 993 (C = C bending), 804 (C–Cl str.) (Figure S6); ^1^H NMR (400 MHz, CDCl_3_) δ ppm: 7.81 (dt, *J* = 6.8, 1.2 Hz, 1H), 7.64 (d, *J* = 9.0 Hz, 1H), 7.58 (td, *J* = 8.3, 6.2, 2.0 Hz, 2 H), 7.43–7.31 (m, 4 H), 7.29 (tt, *J* = 7.0, 6.3, 1.8 Hz, 1H), 7.24 (dd, *J* = 9.0, 1.5 Hz, 1H), 7.15 (d, *J* = 8.8 Hz, 2 H), 6.81 (t, *J* = 7.2 Hz, 2 H), 6.54 (d, *J* = 8.8 Hz, 2 H), 5.06 (s, 2 H), 4.30–4.16 (m, 1H), 3.86 (s, 3 H) (Figure S7); ^13^C NMR (101 MHz, CDCl_3_) δ ppm: 148.0, 136.9, 130.9, 129.8, 128.8, 128.4, 127.7, 127.3, 124.9, 122.7, 120.1, 114.7, 112.2, 111.7, 70.5, 68.2, 55.9, 38.7, 30.3, 28.9, 23.7, 23.0, 14.0, 11.0. (Figure S7); Anal. Calcd for C_27_H_22_CIN_3_O_2_ (455.94): C, 71,13; H, 4.86; N, 9.22%, found: C, 70.95; H, 4.88, N, 9.35%.

#### N-(4-chlorophenyl)−2-(1 H-indol-3-yl)imidazo[1,2-a]pyridin-3-amine (11)

A mixture of 2-aminopyridine (188 mg, 2.0 mmol) and 3-indolcarbaldehyde (290 mg, 2.0 mmol) containing Sc(OTf)_3_ (49 mg, 0.1 mmol) and anhydrous Na_2_SO_4_ (200 mg) in MeOH: dichloromethane (1:1, 10 mL) was stirred under a nitrogen atmosphere for one hour at 50 ^o^C. This was followed by the addition of a solution of 4-chlorophenyl isocyanide (275 mg, 2.0 mmol) in MeOH (2 mL). The mixture was then subjected to the procedure described for compound **9**. The resultant residue was purified by flash chromatography (SiO_2_, dichloromethane/ethyl acetate in 1:1 ratio) to yield the desired compound **11** (224 mg, 31.4%) (Figure S8). MP: 269.2–270.8 °C; IR (cm^−1^): 3357 (N–H str.), 3066 (C–H str. in = C–H), 1624 (C = N str.), 1597 (C = C str.), 1489 (aromatic C = C str.), 1330 (C–N str.), 815 (C–Cl str.) (Figure S9); ^1^H NMR (400 MHz, CDCl_3_) δ ppm: 8.47 (d, *J* = 7.5 Hz, 1H), 8.25 (s, 1H), 7.82 (dt, *J* = 6.7, 1.2 Hz, 1H), 7.69 (dt, *J* = 9.0, 1.1 Hz, 1H), 7.47 (d, *J* = 2.7 Hz, 1H), 7.40–7.35 (m, 1H), 7.24 (t, *J* = 1.5 Hz, 1H), 7.22–7.21 (m, 1H), 7.20 (dt, *J* = 2.7, 1.3 Hz, 1H), 7.16 (d, *J* = 8.8 Hz, 2 H), 6.79 (td, *J* = 6.7, 1.1 Hz, 1H), 6.56 (d, *J* = 8.9 Hz, 2 H), 5.65 (s, 1H) (Figure S10); ^13^C NMR (101 MHz, CDCl_3_) δ ppm: 129.8, 114.6 (Figure S10); Anal. Calcd for C_21_H_15_CIN_4_ (358.82): C,70.29; H, 4.21; N, 15.61%, found: C, 70, 21; H, 4.17; N, 15.52%; HRMS *m/z* calcd for C_21_H_16_CIN_4_ ([M + H]^+^) 359.0985, found 359.1054 (Figure S11).

#### N-(4-chlorophenyl)−2-(2-nitrophenyl)imidazo[1,2-a]pyridin-3-amine (12)

A mixture of 2-aminopyridine (188 mg, 2.0 mmol) and 2-nitrobenzaldehyde (332 mg, 2.2 mmol) containing *p*-toluenesulfonic acid monohydrate (76 mg, 0.4 mmol) and anhydrous Na_2_SO_4_ (200 mg) in MeOH (10 mL) was stirred under a nitrogen atmosphere for one hour at 50 ℃. This was followed by the addition of a solution of 4-chlorophenyl isocyanide (275 mg, 2.0 mmol) in MeOH (2 mL). The mixture was then subjected to the procedure described for **9** except for the reaction time that was 48 h. The resultant residue was purified by flash chromatography (SiO_2_, dichloromethane / ethyl acetate in 7:3 ratio) to provide the compound **12** (293 mg, 40.0%) (Figure S12). MP: 173.3–175.4 °C; IR (cm^−1^): 3140 (N-H str.), 1595 (C = C str.), 1521 (N-O str.), 1491 (aromatic C = C str.), 1347 (C-N str.), 819 (C–Cl str.) (Figure S13); ^1^H NMR (400 MHz, CDCl_3_) ppm: 7.86 (dd, *J* = 8.1, 1.1 Hz, 1H), 7.80 (dt, *J* = 6.9, 1.2 Hz, 1H), 7.73 (dd, *J* = 7.7, 1.4 Hz, 1H), 7.66 (d, *J* = 9.1 Hz, 1H), 7.59 (td, *J* = 7.6, 1.3 Hz, 1H), 7.47 (td, *J* = 8.0, 1.5 Hz, 1H), 7.36–7.27 (m, 1H), 7.1 (d, *J* = 8.9 Hz, 2 H), 6.85 (t, *J* = 6.8, 1.1 Hz, 1H), 6.42 (d, *J* = 8.8 Hz, 2 H), 5.44 (s, 1H) (Figure S14); ^13^C NMR (101 MHz, CDCl_3_) δ ppm: 142.93, 142.86, 132.5, 132.1, 129.6, 128.9, 125.4, 124.4, 122.9, 118.3, 114.5, 112.8, 60.4, 21.1, 14.2, 1.0 (Figure S14); Anal. Calcd for C_19_H_13_CIN_4_O_2_ (364.78): C, 62.56; H, 3.59; N, 15.36%, found: C, 62.41; H, 3.57; N, 15.21%.

#### N-(4-chlorophenyl)−2-(furan-2-yl)imidazo[1,2-a]pyridin-3-amine (13)

A mixture of 2-aminopyridine (188 mg, 2.0 mmol) and 2-furaldehyde (193 mg, 2.0 mmol) containing Sc(OTf)_3_ (49 mg, 0.1 mmol) and anhydrous Na_2_SO_4_ (200 mg) in MeOH (10 mL) was stirred under a nitrogen atmosphere for one hour at 50 ℃. This was followed by the addition of a solution of 4-chlorophenyl isocyanide (303 mg, 2.2 mmol) in MeOH (2 mL). The mixture was then subjected to the procedure described for compound 9. The resultant residue was purified by flash chromatography (SiO_2_, dichloromethane/ethyl acetate in 6:4 ratio) to furnish the title compound 13 (270 mg, 43.6%) (Figure S15). MP: 196.4–198.1 °C; IR (cm^−1^): 3161 (N-H str.), 1599 (C = N str.), 1558 (C = C str.), 1490 (aromatic C = C str.), 1345 (C–N str.), 1260 (C–O str.), 820 (C–Cl str.) (Figure S16); ^1^H NMR (400 MHz, CDCl_3_) ppm: 7.83 (dt, *J* = 6.8, 1.2 Hz, 1H), 7.62 (dt, *J* = 9.1, 1.1 Hz, 1H), 7.46 (dd, *J* = 1.7, 0.8 Hz, 1H), 7.26–7.20 (m, 1H), 7.15 (d, *J* = 8.8 Hz, 2 H), 6.81 (td, *J* = 6.8, 1.2 Hz, 1H), 6.74 (d, *J* = 3.1 Hz, 1H), 6.51 (d, *J* = 8.8 Hz, 2 H), 6.45 (dd, *J* = 3.4, 1.8 Hz, 1H), 5.76 (s, 1H) (Figure S17); ^13^C NMR (101 MHz, CDCl_3_) δ ppm: 142.6, 129.6, 122.7, 115.1, 111.5 (Figure S17); Anal. Calcd for C_17_H_12_CIN_3_O (309.75): C, 65.92; H, 3.91; N, 13.57%. Found: C, 65.78; H, 3.94; N, 13.55%; HRMS *m/z* calcd for C_17_H_11_CIN_3_O ([M-H]^-^) 308.0669, found 308.0591 (Figure S18).

#### N-(4-chlorophenyl)−2-(m-tolyl)imidazo[1,2-a]pyridin-3-amine (14)

A mixture of 2-aminopyridine (188 mg, 2.0 mmol) and *m*-tolualdehyde (240 mg, 2.0 mmol) containing Sc(OTf)_3_ (49 mg, 0.1 mmol) and anhydrous Na_2_SO_4_ (200 mg) in MeOH (10 mL) was stirred under a nitrogen atmosphere for one hour at 50℃. This was followed by the addition of a solution of 4-chlorophenyl isocyanide (303 mg, 2.2 mmol) in MeOH (2 mL). The mixture was then subjected to the procedure described for 9. The resultant residue was purified by flash chromatography (SiO_2_, dichloromethane/ethyl acetate in 8:2 ratio) to afford the desired compound 14 (342 mg, 51.2%) (Figure S19). MP: 183.4–184.3 °C; IR (cm^−1^): 3203 (N–H str.), 2919 (C–H str. in CH_3_), 1598 (C = N str.), 1569 (C = C str.), 1495 (aromatic C = C str.), 1344 (C–N str.), 818 (C–Cl str.) (Figure S20); ^1^H NMR (500 MHz, CDCl_3_) δ ppm: 7.84 (s, 1H), 7.80 (d, *J* = 6.9 Hz, 1H), 7.69 (d, *J* = 7.8 Hz, 1H), 7.63 (d, *J* = 9.1 Hz, 1H), 7.25–7.22 (m, 1H), 7.21 (dd, *J* = 3.6, 1.4 Hz, 1H), 7.15 (d, *J* = 8.9 Hz, 2 H), 7.1 (d, *J* = 7.6 Hz, 1H), 6.79 (td, *J* = 6.8, 1.1 Hz, 1H), 6.51 (d, *J* = 8.7 Hz, 2 H), 5.81 (s, 1H), 2.34 (s, 3 H) (Figure S21); ^13^C NMR (126 MHz, CDCl_3_) δ ppm: 143.3, 142.5, 138.3, 129.7, 128.9, 128.5, 127.7, 125.5, 124.7, 123.9, 122.6, 117.6, 117.4, 114.6, 112.5, 21.5 (Figure S21); Anal. Calcd for C_20_H_16_CIN_3_ (333.81): C, 71.96; H, 4.83; N, 12.59%, found: C, 71.67; H, 85; N, 12.56%; HRMS *m/z* calcd for C_20_H_15_CIN_3_ ([M–H]^-^) 332.1033, found 332.0954 (Figure S22).

#### N-(4-chlorophenyl)−2-(2,3-dimethoxyphenyl)imidazo[1,2-a]pyridin-3-amine (15)

*p*-Toluenesulfonic acid monohydrate (76 mg, 0.4 mmol) was added to a mixture of 2-aminopyridine (188 mg, 2.0 mmol), 2,3-dimethoxybenzaldehyde (332 mg, 2.0 mmol) and anhydrous Na_2_SO_4_ (200 mg) in MeOH (10.0 mL). After stirring under a nitrogen atmosphere at 50 °C for one hour, a solution of 4-chlorophenyl isocyanide (303 mg, 2.0 mmol) in MeOH (2 mL) was added. The mixture was then subjected to the procedure described for compound 9. The residue was purified by flash column chromatography on silica gel in a 1:1 ratio of (dichloromethane /ethyl acetate in 1:1 ratio) to afford the title compound 15, which was further recrystallized from 95% of ethanol (372 mg, 49%) (Figure S23). MP: 220–221 °C; IR (cm^−1^): 2879 (C-H str. in CH_3_), 1597 (C = N str.), 1580 (C = C str.), 1490 (aromatic C = C str.), 1348 (C-N str.), 1262 (C–O str.), 819 (C–Cl str.) (Figure S24); ^1^H NMR (500 MHz, CDCl_3_) ppm: 7.72–7.65 (m, 2 H), 7.46 (dd, *J* = 8.0, 1.5 Hz, 1H), 7.23 (d, *J* = 8.3 Hz, 1H), 7.19 (dd, *J* = 8.0 Hz, 1H), 7.07 (d, *J* = 8.8 Hz, 2 H), 6.95 (dd, *J* = 8.1, 1.5 Hz, 1H), 6.91 (s, 1H), 6.82 (dd, *J* = 7.3, 1H), 6.34 (d, *J* = 8.9, 2 H), 3.92 (s, 3 H), 3.66 (s, 3 H) (Figure S25); ^13^C NMR (126 MHz, CDCl_3_) δ ppm: 152.6, 145.9, 142.7, 142.3, 129.2, 125.0, 124.6, 123.3, 123.0, 121.3, 117.8, 115.8, 112.1, 111.9, 61.6, 55.8, 31.6, 30.9, 22.7, 15.3, 14.1 (Figure S25); Anal. Calcd for C_21_H_18_CIN_3_O_2_ (379.84): C, 66.40; H, 4.78; N, 11.06%, found: C, 66.32; H, 4.79; N, 11.10%.

#### N-(4-chlorophenyl)−2-(naphthalen-2-yl)imidazo[1,2-a]pyridin-3-amine (16)

A mixture of 2-aminopyridine (188 mg, 2.0 mmol) and 2-naphthaldehyde (312 mg, 2.0 mmol) containing *p*-toluenesulfonic acid monohydrate (76 mg, 0.4 mmol) and sodium sulphate (200 mg) in MeOH (10 mL) was stirred under a nitrogen atmosphere for one hour at 50 ℃. Then, a solution of 4-chlorophenyl isocyanide (303 mg, 2.2 mmol) in MeOH (2 mL) was added, and the mixture was subjected to the procedure described for **9**. Purification by flash column chromatography (SiO_2_, dichloromethane/ethyl acetate in 6:4 ratio), followed by recrystallization from 95% ethanol yielded **16** as white crystals (354 mg, 47.9%) (Figure S26). MP: 225.7–226.7 °C; IR (cm^−1^): 3198 (N-H str.), 3073 (C-H str. in = C-H), 1600 (C = N str.), 1569 (C = C str.), 1491 (aromatic C = C str.), 1344 (C–N str.), 815 (C–Cl str.) (Figure S27); ^1^H NMR (500 MHz, CDCl_3_) δ ppm: 8.47 (s, 1H), 8.08 (dd, *J* = 8.6, 1.6 Hz, 1H), 7.87–7.74 (m, 4 H), 7.66 (d, *J* = 9.0 Hz, 1H), 7.46 (dt, *J* = 9.4, 3.1 Hz, 2 H), 7.26–7.21 (m, 1H), 7.18 (d, *J* = 8.8 Hz, 2 H), 6.79 (t, *J* = 6.8, 1.1 Hz, 1H), 6.57 (d, *J* = 8.7 Hz, 2 H), 5.71 (s, 1H) (Figure S28); ^13^C NMR (126 MHz, CDCl_3_) δ ppm: 143.3, 142.9, 133.4, 133.0, 129.8, 128.5, 128.2, 127.6, 126.2, 125.4, 124.78, 124.65, 122.6, 117.86, 117.69, 114.7, 112.5 (Figure S28); Anal. Calcd for C_23_H_16_CIN_3_ (369.85): C, 74.69; H, 4.36; N, 11.36% found: C, 74.38; H, 4.32; N, 11.34%.

***N-(4-chlorophenyl)−2-(thiophen-3-yl)imidazo[1***,***2-a]pyridin-3-amine (17)***

A mixture of 2-aminopyridine (188 mg, 2.0 mmol) and 3-thiophenecarboxaldehyde (230 mg, 2.0 mmol) containing *p*-toluenesulfonic acid monohydrate (76 mg, 0.4 mmol) and sodium sulphate (200 mg) in MeOH (10 mL) was stirred under a nitrogen atmosphere for one hour at 50 ℃. Then, a solution of 4-chlorophenyl isocyanide (303 mg, 2.2 mmol) in MeOH (2 mL) was added, and the mixture was subjected to the procedure described for **9**. The residue was recrystallized with 95% ethanol twice, yielded compound **17** as hairy crystals (214 mg, 32.8%) (Figure S29). MP: 245.8–246.9 °C; IR (cm^−1^): 3209 (N-H str.), 1631 (C = N str.), 1595 (C = C str.), 1492 (aromatic C = C str.), 1345 (C–N str.), 1093 (C = S str.), 816 (C–Cl str.) (Figure S30); ^1^H NMR (500 MHz, CDCl_3_) δ ppm: 7.83 (d?, *J* = 6.9 Hz, 1H), 7.76 (dd, *J* = 3.0, 1.1 Hz, 1H), 7.67–7.57 (m, 2 H), 7.33 (dd, *J* = 5.2, 3.0 Hz, 1H), 7.24 (ddd, *J* = 9.1, 6.8, 1.3 Hz, 1H), 7.16 (d, *J* = 8.8 Hz, 2 H), 6.80 (dd, *J* = 6.8, 1.1 Hz, 1H), 6.53 (d, *J* = 8.9 Hz, 2 H), 5.58 (s, 1H) (Figure S31); ^13^C NMR (126 MHz, CDCl_3_) δ ppm: 143.3, 142.9, 136.8, 134.4, 129.8, 126.4, 125.91, 125.26, 124.8, 122.69, 122.44, 117.6, 116.8, 114.5, 112.4 (Figure S31); Anal. Calcd for C_17_H_12_CIN_3_S (325.81): C, 62.67; H, 3.71; N, 12.90%, found: C, 62.26; H, 3.72; N, 12.92%.

#### N-(4-chlorophenyl)−2-(2,4-difluorophenyl)imidazo[1,2-a]pyridin-3-amine (18)

A mixture of 2-aminopyridine (188 mg, 2.0 mmol), and 2,4-difluorobenzaldehyde (284 mg, 2.0 mmol) containing *p*-toluenesulfonic acid monohydrate (76 mg, 0.4 mmol) in MeOH (10 mL) was stirred under a nitrogen atmosphere for one hour at 50 °C. This was followed by the addition of a solution of 4-chlorophenyl isocyanide (303 mg, 2.2 mmol) in MeOH (2 mL). The mixture was then subjected to the procedure described for **9.** The resultant residue was purified by flash chromatography (SiO_2_, dichloromethane/ethyl acetate in 8:2 ratio) to yield the desired compound **18** (259 mg, 36.6%) (Figure S32). MP: 154.8–156.9 °C; IR (cm^−1^): 3160 (N-H str.), 3080 (C–H str. in = C–H), 1597 (C = N str.), 1574 (C = C str.), 1491 (aromatic C = C str.), 1266 (C-N str.), 1141 (C-F str.); 970 (C = C bending), 818 (C-Cl str.) (Figure S33); ^1^H NMR (400 MHz, CDCl_3_) δ ppm: 7.83 (d, *J* = 8.5 Hz, 1H), 7.8 (d, *J* = 8.5 Hz, 1H), 7.66 (d, *J* = 9.1 Hz, 1H), 7.27 (ddd, *J* = 9.0, 6.7, 1.2 Hz, 1H), 7.11 (d, *J* = 8.8 Hz, 2 H), 6.96 (td, *J* = 8.2, 2.7 Hz, 1H), 6.91–6.84 (m, 1H), 6.83 (dd, *J* = 6.7, 2.4 Hz, 1H), 6.41 (d, *J* = 8.9 Hz, 2 H), 5.76 (d, *J* = 2.7 Hz, 1H) (Figure S34); ^13^C NMR (101 MHz, CDCl3) δ ppm: 164.21, 164.08, 161.72, 161.60, 161.17, 161.05, 158.69, 158.57, 143.18, 143.0, 134.0, 132.28, 132.23, 132.19, 132.13, 129.5, 125.2, 124.7, 123.0, 119.7, 118.0, 114.8, 112.51, 112.11, 112.08, 111.90, 111.87, 104.46, 104.20, 103.9 (Figure S34); ^19^F NMR (377 MHz, CDCl3) δ ppm: − 109.16, − 110.27 (Figure S35); Anal. Calcd for C_19_H_12_CIF_2_N_2_ (355.77): C, 64.14; H, 3.40; N, 11.81%, found: C, 64.11; H, 3.41; N, 11.83%.

#### N-(4-chlorophenyl)−2-(4-(trifluoromethyl)phenyl)imidazo[1,2-a]pyridin-3-amine (19)

A mixture of 2-aminopyridine (188 mg, 2.0 mmol), 4-trifluoromethyl-benzaldehyde (348 mg, 2.0 mmol), *p*-toluenesulfonic acid monohydrate (76 mg, 0.4 mmol) and Na_2_SO_4_ (200 mg) in MeOH (10 mL) was stirred under a nitrogen atmosphere for one hour at 50 ℃. This was followed by the addition of 4-chlorophenyl isocyanide (280 mg, 2.0 mmol). The mixture was then subjected to the procedure described for **9**. The residue was purified by flash column chromatography (SiO_2_, dichloromethane/ethyl acetate in 7:3 ratio) to yield the desired compound **19** as pale-yellow solid (240 mg, 30.9%) (Figure S36). MP: 197.5–199 °C; IR (cm^−1^): 3147 (N-H str.), 3069 (C–H str. in = C–H), 1621 (C = N str.), 1599 (C = C str.), 1490 (aromatic C = C str.), 1323 (C–N str.), 1120 (C–F str.), 825 (C–Cl str.) (Figure S37); ^1^H NMR (400 MHz, CDCl_3_) ppm: 8.11 (d, *J* = 8.1 Hz, 2 H), 7.83 (dt, *J* = 6.8, 1.2 Hz, 1H), 7.66 (dt, *J* = 9.1, 1.1 Hz, 1H), 7.62 (d, *J* = 8.7 Hz, 2 H), 7.29 (dd, *J* = 9.1, 6.7, 1.3 Hz, 1H), 7.18 (d, *J* = 8.8 Hz, 2 H), 6.83 (td, *J* = 7.9, 6.8, 1.1 Hz, 1H), 6.54 (d, *J* = 8.8 Hz, 2 H), 5.69 (s, 1H) (Figure S38); ^13^C NMR (101 MHz, CDCl_3_) δ ppm: 142.9, 136.6, 129.92, 129.55, 129.23, 127.1, 125.84, 125.59, 125.55, 125.15, 122.7, 118.3, 118.0, 114.6, 112.9 (Figure S38); ^19^F NMR (377 MHz, CDCl3) δ ppm: −62.58 (Figure S39); Anal. Calcd for C_20_H_13_CIF_3_N_3_ (387.07): C, 61.95; H, 3.38; N, 10.84%, found: C, 61.82; H, 3.42; N, 10.81%.

### XRD-Analysis

Using Crystal Explorer 3.1, HSA computations were performed [29]. The XRD-crystallographic data for the desired ligand was acquired at a temperature of 296 °K using the CrysalisPro program [30]. This was accomplished with Mo radiation (wavelength = 0.71073) and a Gemini kappa-geometry diffractometer, especially a Rigaku XtaLAB P200K fitted with an Atlas CCD detector. SHELXT [31] was used to make the structural determination. Table S2 displays the parameters and crystallographic data used to refine the structure of compound **15.**

### Biological activity

#### Anticancer activity

The synthesized compounds were assessed for their cytotoxic effects on three different cancer cell lines: the MCF-7 human breast cancer cell line (ATCC number: HTB-22), the HT-29 human colorectal adenocarcinoma cell line [HTB-38; American Type Culture Collection (ATCC), Manassas, VA, USA], and the B16F10 murine melanoma cancer cell line [CRL-6475; American Type Culture Collection (ATCC), Manassas, VA, USA], as well as the normal cell line mouse Embryonic Fibroblast-1 cell line (MEF-1; ATCC CRL-2214). The cell line was cultivated in high-glucose Dulbecco’s Modified Eagle Medium (DMEM) that is supplemented with L-glutamine, phenol red (Fuji Film Wako, Osaka, Japan), 10% fetal bovine serum (FBS; G.E. Healthcare, Chicago, IL, USA), and 1% penicillin/streptomycin (P/S) (Nacalai Tesque, Kyoto, Japan). Exponentially growing cells (1 × 10^5^ cells/well) were seeded in a 24-well plate with sterile-filtered DMEM medium at 37 °C under 5% CO_2_ and incubated overnight. The cells were then treated with different concentrations of test compounds (5, 10, 50, and 100 µg/mL) and incubated for 24 h in a humidified atmosphere. After 24 h of incubation, viable cells were counted in each well using the Trypan blue exclusion test. All compounds were initially dissolved in DMSO, and the final concentration of DMSO was less than 1% in all of the considered concentrations of the applied compounds. The IC_50_ values from at least two independent experiments were compared with the control and expressed as the mean ± SD. The analysis of two groups was performed using the student t-test, and P-values < 0.05 were statistically significant.

## Supplementary Information

Supplementary Material 1.

## Data Availability

The data underlying this study are available in the published article and its online supplementary material. Supporting information is freely accessible online and includes the supplementary crystallographic data for this paper (CCDC No. 2401478). These data can be obtained free of charge via https://www.ccdc.cam.ac.uk/structures/Search? access=referee&ccdc=2401478&Author=Saki+Raheem, by emailing data_request@ccdc.cam.ac.uk, or by contacting The Cambridge Crystallographic Data Centre, 12 Union Road, Cambridge CB2 1EZ, UK; fax: +44-1223-336033.

## Abbreviations

IR: Infrared
FTIR: Fourier-transform infrared spectroscopy
HPLC: High-performance liquid chromatography
HRMS: High-resolution mass spectrometry
^1^H NMR: Proton nuclear magnetic resonance
^13^C NMR: Carbon nuclear magnetic resonance
DFT: Density functional theory
μM: Micromolar
°C: Degree centigrade
h: Hour
g: Gram
mg: Milligram
mL: Millilitre
μL: Microliter
DMSO: Dimethylsulfoxide
XDR/HSA: X-ray Diffraction/Human Serum Albumin
