# Supplementary material for: Synthesis and biological activities of 3-aminoimidazo[1,2-α]pyridine compounds
Source: BMC Chem. 2025 Feb 22;19(1):48. doi: 10.1186/s13065-025-01412-6 (PMC11847391; doi:10.1186/s13065-025-01412-6)
Supplement: Supplementary file 1 — Supplementary Material 1. [file 13065_2025_1412_MOESM1_ESM.docx]

**Supplementary Information**

**Synthesis and Biological Activities of 3-Aminoimidazole[1,2-α]pyridine Analogues.**

Isra’ Al-Qadi^1^, Michel Hanania^2^, Ismail Warad^1^, Nisreen Al-Hajj^1^, Rand Hazzam^1^, Yousef Salama^3^, Saki Raheem^4,^*, Nawaf Al-Maharik^1,^ *

^1^Department of Chemistry, Faculty of Science, An-Najah National University, Nablus, 00970, Palestine

^2^Department of Chemistry, Faculty of Applied Sciences, Technology and Engineering, Bethlehem University, Bethlehem, 00970, Palestine

^3^Department of Biomedical Sciences, Faculty of Medicine and Health Sciences, An-Najah National University, Nablus, 00970, Palestine

^4^School of Life Sciences, University of Westminster, 115 New Cavendish Street, London, W1W 6UW, United Kingdom

Corresponding authors:

✉︎ Saki Raheem

[s.raheem@westminster.ac.uk](mailto:s.raheem@westminster.ac.uk)

✉︎ Nawaf Al-Maharik

[n.maharik@najah.edu](mailto:n.maharik@najah.edu)

**Table S1.** XDR/DFT-bond lengths (Å) and angle (^o^) compound **15.**

| **No.** | **Bonds** | | **XRD** | **DFT** | **No** | **Angles** | | | **XRD** | **DFT** |
| --- | --- | --- | --- | --- | --- | --- | --- | --- | --- | --- |
| 1 | Cl1 | C19 | 1.739(2) | 1.76 | 1 | C9 | O1 | C14 | 115.1(2) | 109.47 |
| 2 | O1 | C9 | 1.379(3) | 1.43 | 2 | C2 | N1 | C3 | 130.4(2) | 130.13 |
| 3 | O1 | C14 | 1.420(4) | 1.43 | 3 | C2 | N1 | C7 | 107.0(2) | 107.71 |
| 4 | N1 | C2 | 1.390(3) | 1.4492 | 4 | C3 | N1 | C7 | 122.7(2) | 122.16 |
| 5 | N1 | C3 | 1.369(3) | 1.327 | 5 | N2 | C1 | C2 | 111.5(2) | 109.39 |
| 6 | N1 | C7 | 1.378(3) | 1.4338 | 6 | N2 | C1 | C8 | 121.0(2) | 125.3 |
| 7 | C1 | N2 | 1.375(3) | 1.49 | 7 | C2 | C1 | C8 | 127.5(2) | 125.31 |
| 8 | C1 | C2 | 1.359(3) | 1.4009 | 8 | C1 | N2 | C7 | 105.3(2) | 106.03 |
| 9 | C1 | C8 | 1.486(3) | 1.54 | 9 | C10 | O2 | C15 | 117.7(3) | 109.48 |
| 10 | N2 | C7 | 1.332(3) | 1.3505 | 10 | N1 | C2 | C1 | 105.4(2) | 105.95 |
| 11 | O2 | C10 | 1.364(4) | 1.43 | 11 | N1 | C2 | N3 | 122.3(2) | 127.02 |
| 12 | O2 | C15 | 1.429(4) | 1.4299 | 12 | C1 | C2 | N3 | 132.2(2) | 127.02 |
| 13 | C2 | N3 | 1.386(3) | 1.47 | 13 | N1 | C3 | C4 | 118.5(2) | 118.12 |
| 14 | C3 | C4 | 1.345(4) | 1.3699 | 14 | C2 | N3 | C16 | 123.4(2) | 109.48 |
| 15 | N3 | C16 | 1.378(3) | 1.4699 | 15 | C3 | C4 | C5 | 120.7(3) | 121.26 |
| 16 | C4 | C5 | 1.397(4) | 1.4262 | 16 | N1 | C7 | N2 | 110.9(2) | 110.92 |
| 17 | C7 | C6 | 1.408(3) | 1.3864 | 17 | N1 | C7 | C6 | 118.3(2) | 120.2 |
| 18 | C6 | C5 | 1.355(4) | 1.366 | 18 | N2 | C7 | C6 | 130.9(2) | 128.88 |
| 19 | C8 | C9 | 1.374(3) | 1.4013 | 19 | C7 | C6 | C5 | 118.7(3) | 117.37 |
| 20 | C8 | C13 | 1.392(4) | 1.4014 | 20 | C4 | C5 | C6 | 121.2(3) | 120.89 |
| 21 | C9 | C10 | 1.406(3) | 1.4014 | 21 | C1 | C8 | C9 | 120.1(2) | 120.01 |
| 22 | C10 | C11 | 1.374(5) | 1.4014 | 22 | C1 | C8 | C13 | 120.2(2) | 120 |
| 23 | C13 | C12 | 1.376(5) | 1.4014 | 23 | C9 | C8 | C13 | 119.7(2) | 120 |
| 24 | C12 | C11 | 1.380(5) | 1.4014 | 24 | O1 | C9 | C8 | 119.9(2) | 120 |
| 25 | C20 | C21 | 1.381(3) | 1.4013 | 25 | O1 | C9 | C10 | 119.6(2) | 120 |
| 26 | C20 | C19 | 1.376(4) | 1.4014 | 26 | C8 | C9 | C10 | 120.3(2) | 120 |
| 27 | C21 | C16 | 1.396(3) | 1.4015 | 27 | O2 | C10 | C9 | 115.1(2) | 120 |
| 28 | C19 | C18 | 1.370(4) | 1.4015 | 28 | O2 | C10 | C11 | 125.6(3) | 120 |
| 29 | C18 | C17 | 1.386(4) | 1.4013 | 29 | C9 | C10 | C11 | 119.2(3) | 120 |
| 30 | C17 | C16 | 1.396(3) | 1.4014 | 30 | C8 | C13 | C12 | 119.9(3) | 120 |
|  |  |  |  |  | 31 | C13 | C12 | C11 | 120.5(3) | 120 |
|  |  |  |  |  | 32 | C10 | C11 | C12 | 120.4(3) | 120 |
|  |  |  |  |  | 33 | C21 | C20 | C19 | 120.2(2) | 120 |
|  |  |  |  |  | 34 | C20 | C21 | C16 | 120.9(2) | 120 |
|  |  |  |  |  | 35 | Cl1 | C19 | C20 | 119.8(2) | 120 |
|  |  |  |  |  | 36 | Cl1 | C19 | C18 | 120.0(2) | 120 |
|  |  |  |  |  | 37 | C20 | C19 | C18 | 120.2(2) | 120 |
|  |  |  |  |  | 38 | C19 | C18 | C17 | 120.0(2) | 120 |
|  |  |  |  |  | 39 | C18 | C17 | C16 | 120.9(2) | 120 |
|  |  |  |  |  | 40 | N3 | C16 | C21 | 122.8(2) | 120 |
|  |  |  |  |  | 41 | N3 | C16 | C17 | 119.5(2) | 120 |
|  |  |  |  |  | 42 | C21 | C16 | C17 | 117.7(2) | 120 |

**Table S2.** Parameters and crystallographic data used to refine the structure of analogue **15.**

| **Chemical formula** | [**C_21_H_18_ClN_3_O_2_**](file:///C:\Users\Surface%20pro\Downloads\IQ09%20_chemical_formula_sum) |
| --- | --- |
| *M*_r_ | [379.83](file:///C:\Users\Surface%20pro\Downloads\IQ09%20_chemical_formula_weight) |
| Crystal system, space group | [Orthorhombic](file:///C:\Users\Surface%20pro\Downloads\IQ09%20_space_group_crystal_system), [P2_1_2_1_2_1_](file:///C:\Users\Surface%20pro\Downloads\IQ09%20_space_group_name_H-M_alt) |
| Temperature (K) | [296](file:///C:\Users\Surface%20pro\Downloads\IQ09%20_cell_measurement_temperature) |
| *a*, *b*, *c* (Å) | [10.2676 (3)](file:///C:\Users\Surface%20pro\Downloads\IQ09%20_cell_length_a), [11.1678 (3)](file:///C:\Users\Surface%20pro\Downloads\IQ09%20_cell_length_b), [16.8317 (5)](file:///C:\Users\Surface%20pro\Downloads\IQ09%20_cell_length_c) |
| *V* (Å^3^) | [1930.03 (10)](file:///C:\Users\Surface%20pro\Downloads\IQ09%20_cell_volume) |
| *Z* | [4](file:///C:\Users\Surface%20pro\Downloads\IQ09%20_cell_formula_units_Z) |
| Radiation type | [Cu Kα](file:///C:\Users\Surface%20pro\Downloads\IQ09%20_diffrn_radiation_type) |
| µ (mm^−1^) | [1.92](file:///C:\Users\Surface%20pro\Downloads\IQ09%20_exptl_absorpt_coefficient_mu) |
| Crystal size (mm) | [0.25](file:///C:\Users\Surface%20pro\Downloads\IQ09%20_exptl_crystal_size_max) × [0.20](file:///C:\Users\Surface%20pro\Downloads\IQ09%20_exptl_crystal_size_mid) × [0.12](file:///C:\Users\Surface%20pro\Downloads\IQ09%20_exptl_crystal_size_min) |
| Diffractometer | [Bruker APEX-II CCD](file:///C:\Users\Surface%20pro\Downloads\IQ09%20_diffrn_measurement_device_type) |
| Absorption correction | [Multi-scan](file:///C:\Users\Surface%20pro\Downloads\IQ09%20_exptl_absorpt_correction_type)  [SADABS2016/2 - Bruker AXS area detector scaling and absorption correction](file:///C:\Users\Surface%20pro\Downloads\IQ09%20_exptl_absorpt_process_details) |
| *T*_min_, *T*_max_ | [0.55](file:///C:\Users\Surface%20pro\Downloads\IQ09%20_exptl_absorpt_correction_T_min), [0.80](file:///C:\Users\Surface%20pro\Downloads\IQ09%20_exptl_absorpt_correction_T_max) |
| No. of measured, independent and observed [[I > 2σ(I)](file:///C:\Users\Surface%20pro\Downloads\IQ09%20_reflns_threshold_expression)] reflections | [14129](file:///C:\Users\Surface%20pro\Downloads\IQ09%20_diffrn_reflns_number), [3382](file:///C:\Users\Surface%20pro\Downloads\IQ09%20_reflns_number_total), [3213](file:///C:\Users\Surface%20pro\Downloads\IQ09%20_reflns_number_gt) |
| *R*_int_ | [0.039](file:///C:\Users\Surface%20pro\Downloads\IQ09%20_diffrn_reflns_av_R_equivalents) |
| (sin θ/λ)_max_ (Å^−1^) | 0.595 |
| *R*[*F*^2^ > 2σ(*F*^2^)], *wR*(*F*^2^), *S* | [0.033](file:///C:\Users\Surface%20pro\Downloads\IQ09%20_refine_ls_R_factor_gt), [0.089](file:///C:\Users\Surface%20pro\Downloads\IQ09%20_refine_ls_wR_factor_ref), [1.02](file:///C:\Users\Surface%20pro\Downloads\IQ09%20_refine_ls_goodness_of_fit_ref) |
| No. of reflections | [3382](file:///C:\Users\Surface%20pro\Downloads\IQ09%20_refine_ls_number_reflns) |
| No. of parameters | [244](file:///C:\Users\Surface%20pro\Downloads\IQ09%20_refine_ls_number_parameters) |
| Absolute structure parameter | [0.022 (8)](file:///C:\Users\Surface%20pro\Downloads\IQ09%20_refine_ls_abs_structure_Flack) |
| Δρ_max_, Δρ_min_ (e Å^−3^) | [0.18](file:///C:\Users\Surface%20pro\Downloads\IQ09%20_refine_diff_density_max), [−0.30](file:///C:\Users\Surface%20pro\Downloads\IQ09%20_refine_diff_density_min) |

**Figure S1.** HPLC chromatogram of compound **9**.


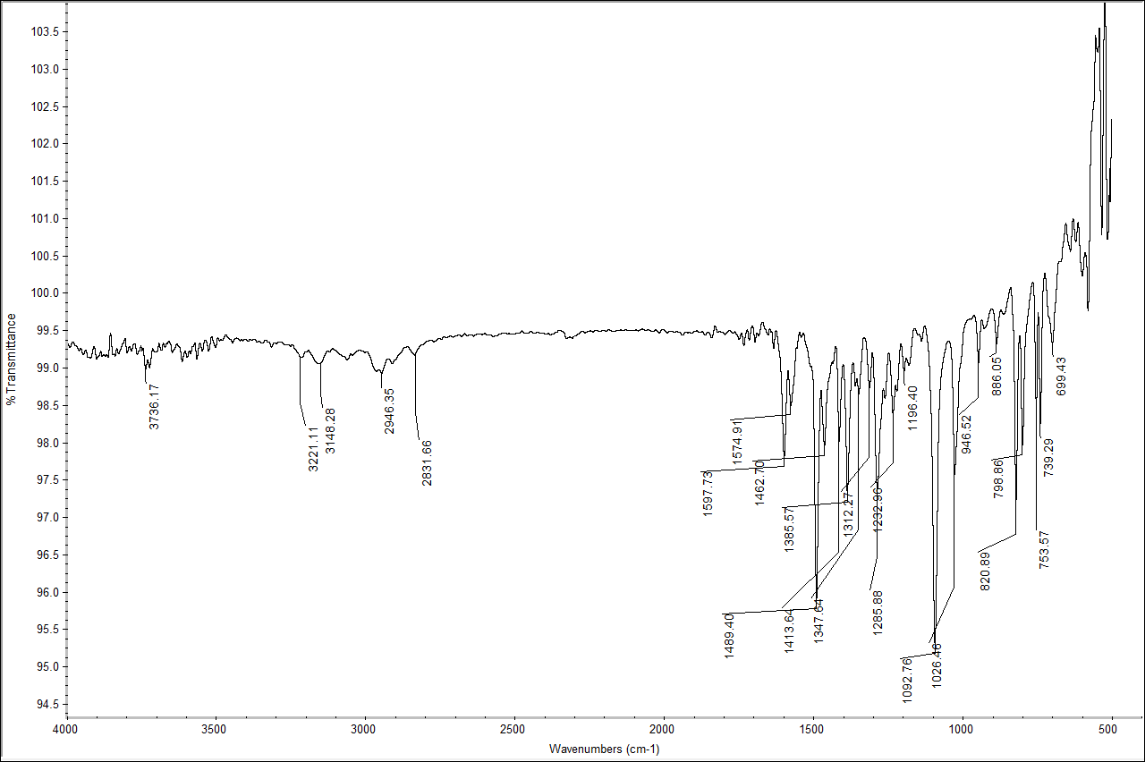


**Figure S2.** IR spectrum of compound **9**.

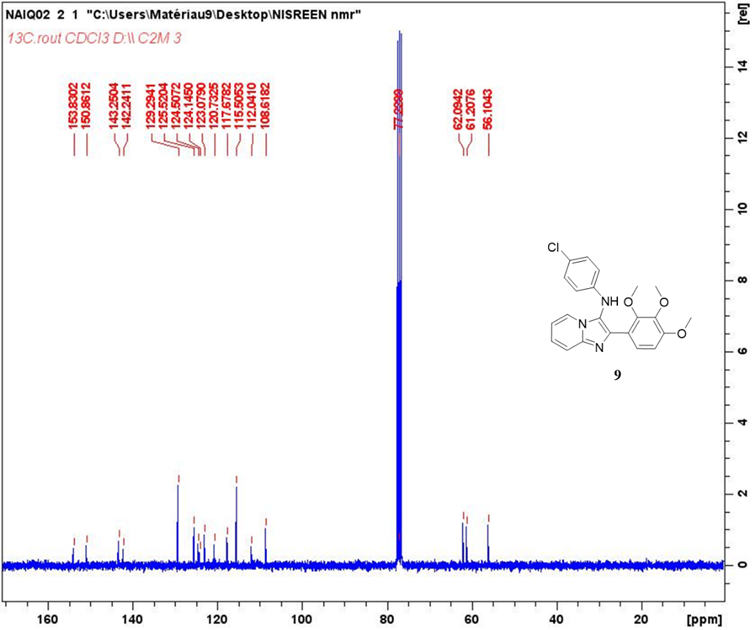


**Figure S3.** ^1^H and ^13^C spectrum of compound **9**.

**Figure S4.** Mass spectrum of compound **9**.

**Figure S5.** HPLC chromatogram of compound **10**.


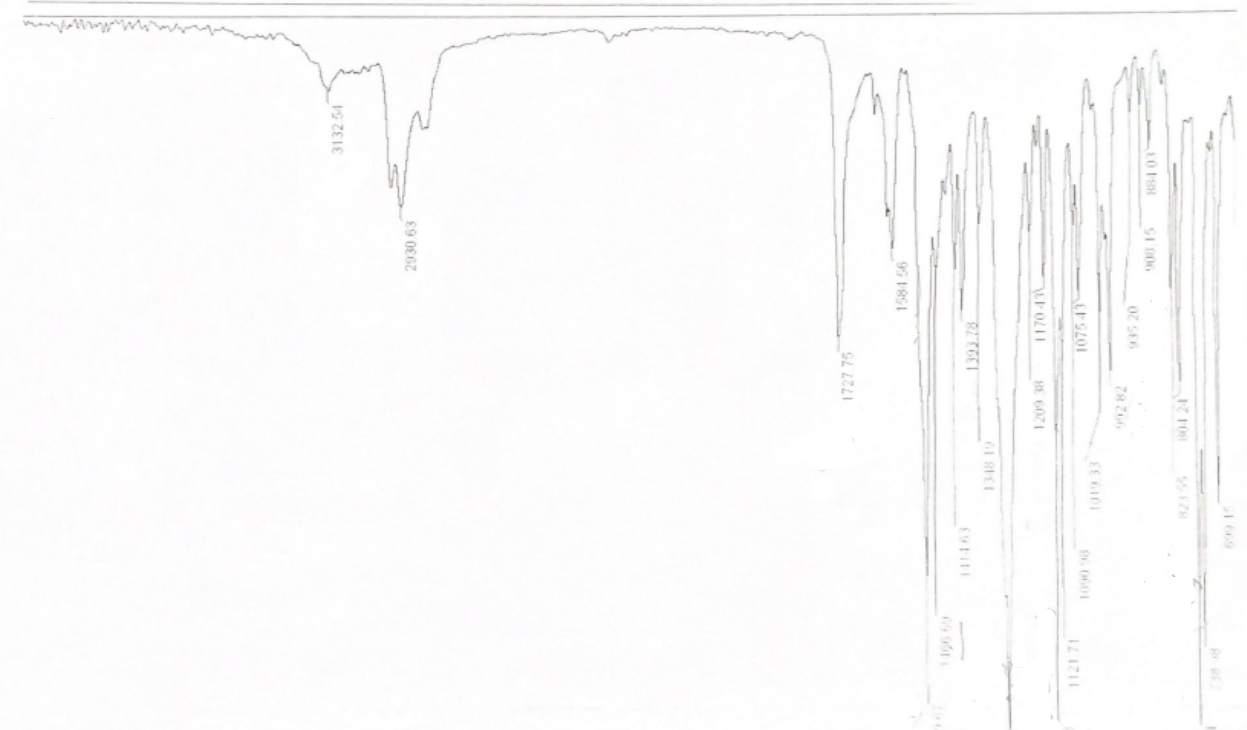


**Figure S6.** IR spectrum of compound **10**.

**Figure S7.** ^1^H and ^13^C spectrum of compound **10**.

**Figure S8.** HPLC chromatogram of compound **11**.

*
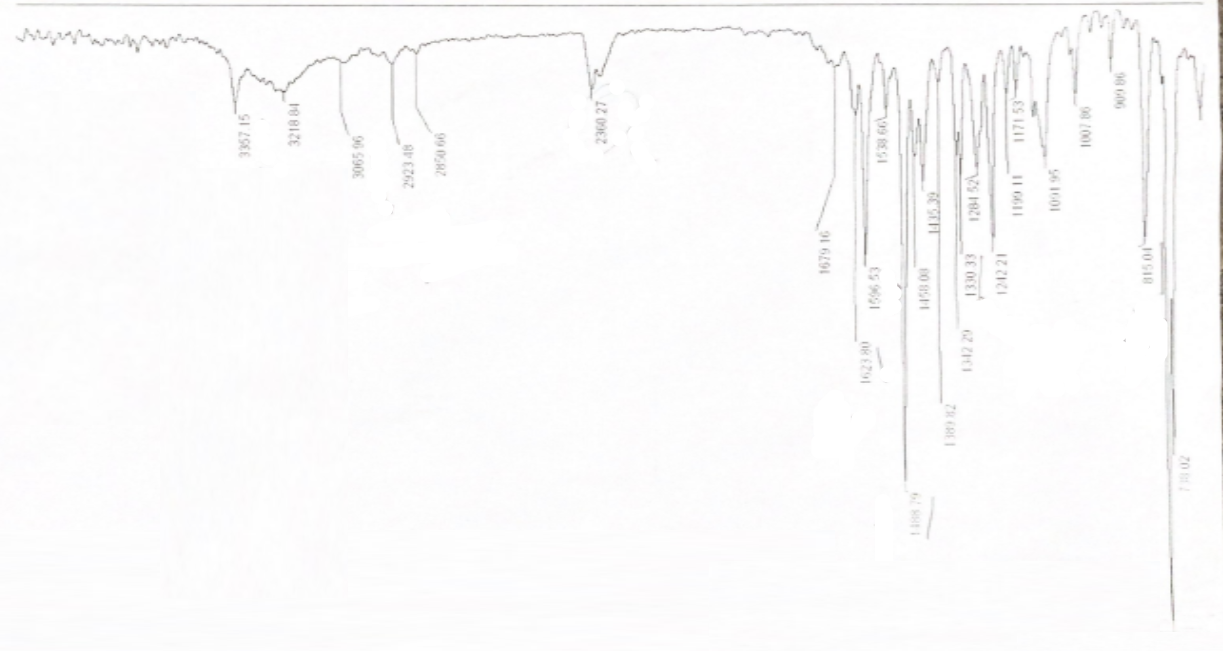
*

**Figure S9.** IR spectrum of compound **11**.

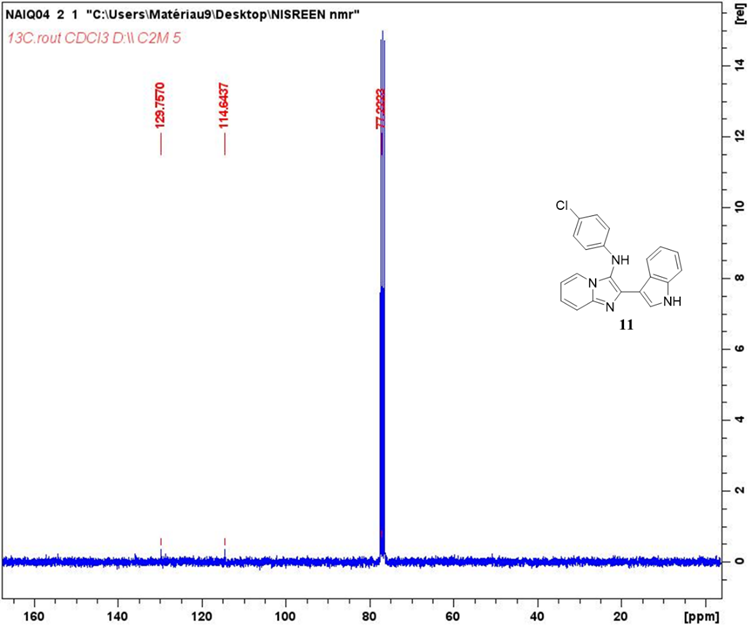


**Figure S10.** ^1^H and ^13^C spectrum of compound **11**.

**Figure S11.** Mass spectrum of compound **11**.

**Figure S12.** HPLC chromatogram of compound **12**.

*
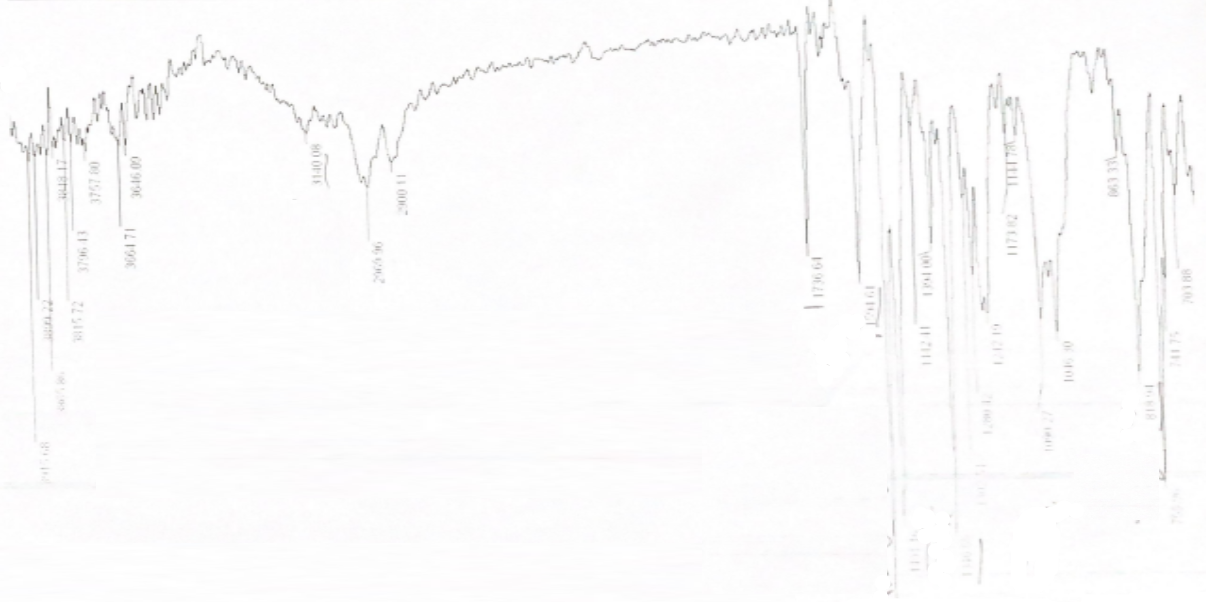
*

**Figure S13.** IR spectrum of compound **12**.

**Figure S14.** ^1^H and ^13^C spectrum of

**Figure S15.** HPLC chromatogram of compound **13**.


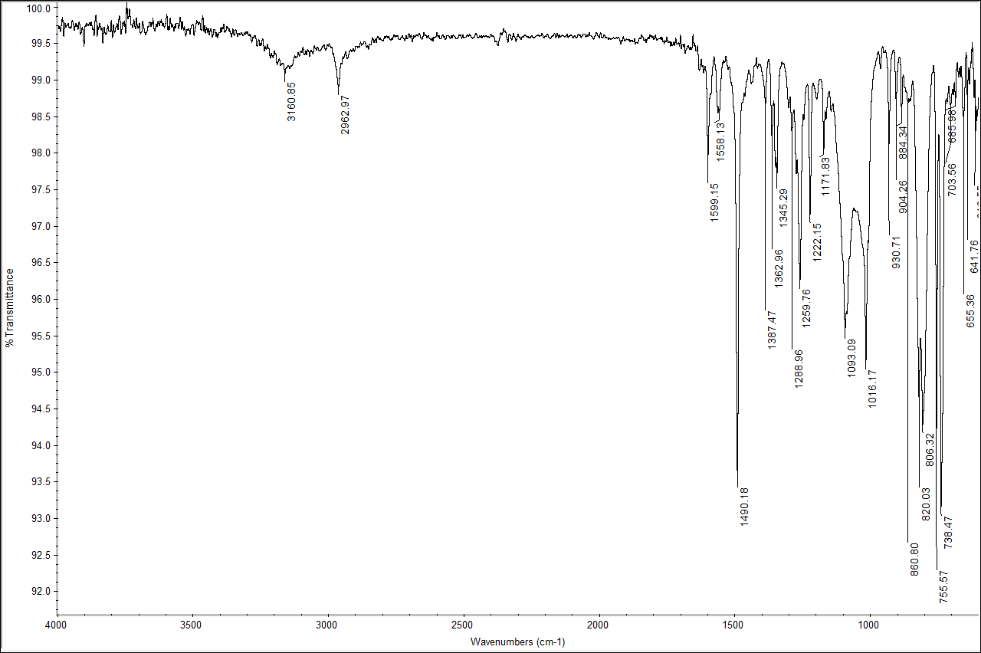


**Figure S16.** IR spectrum of compound **13**.

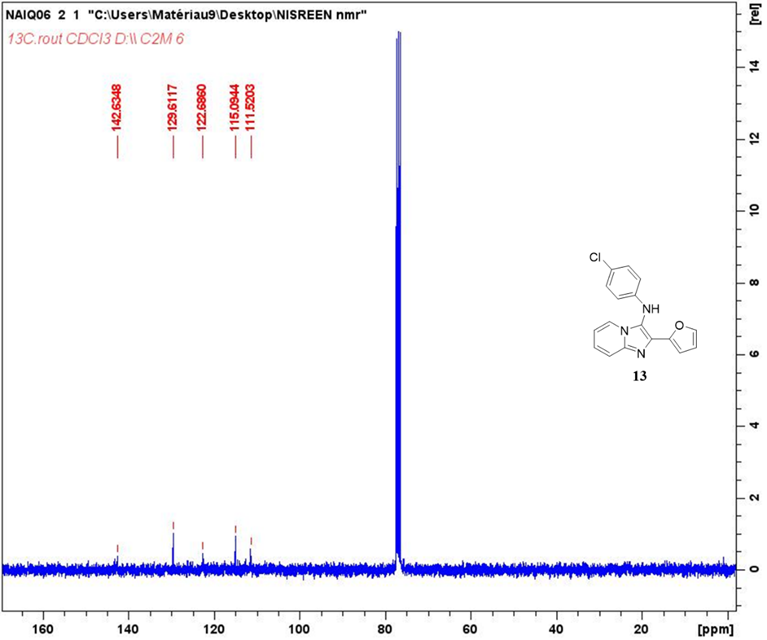


**Figure S17.** ^1^H and ^13^C spectrum of compound **13**.

**Figure S18.** Mass spectrum of compound **13**.

**Figure S19.** HPLC chromatogram of compound **14**.


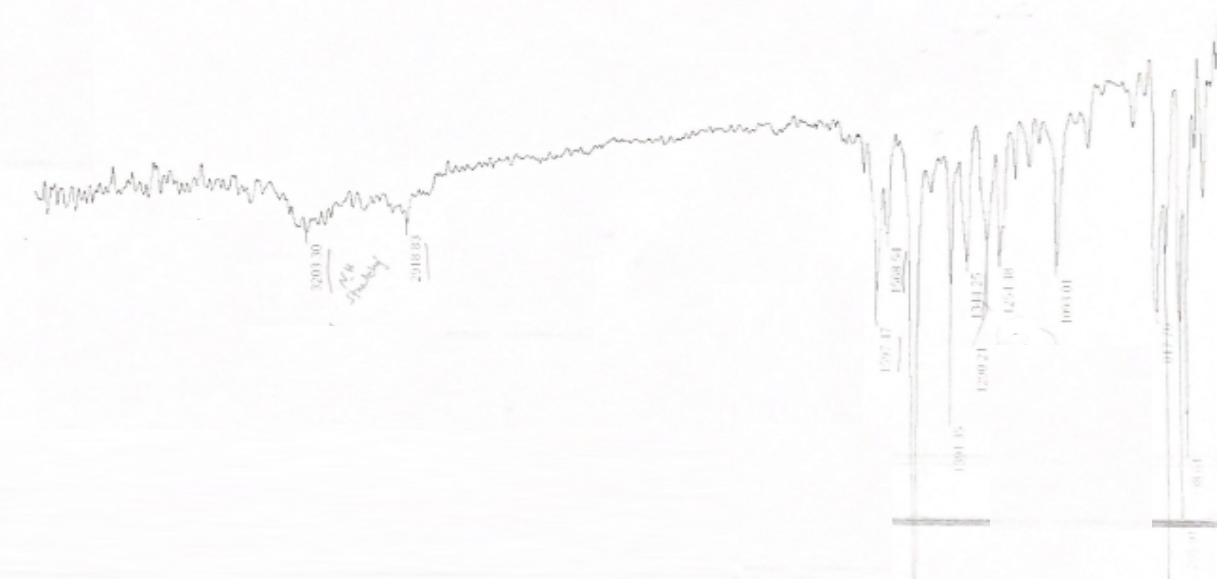


**Figure S20.** IR spectrum of compound **14**.

**Figure S21.** ^1^H and ^13^C spectrum of compound **14**.

**Figure S22.** Mass spectrum of compound **14**.


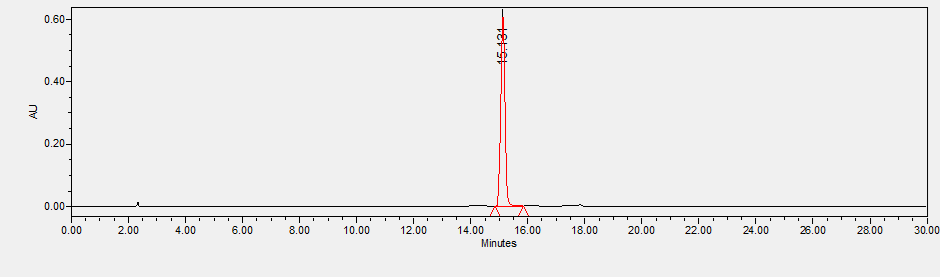


**Figure S23.** HPLC chromatogram of compound **15**.

*
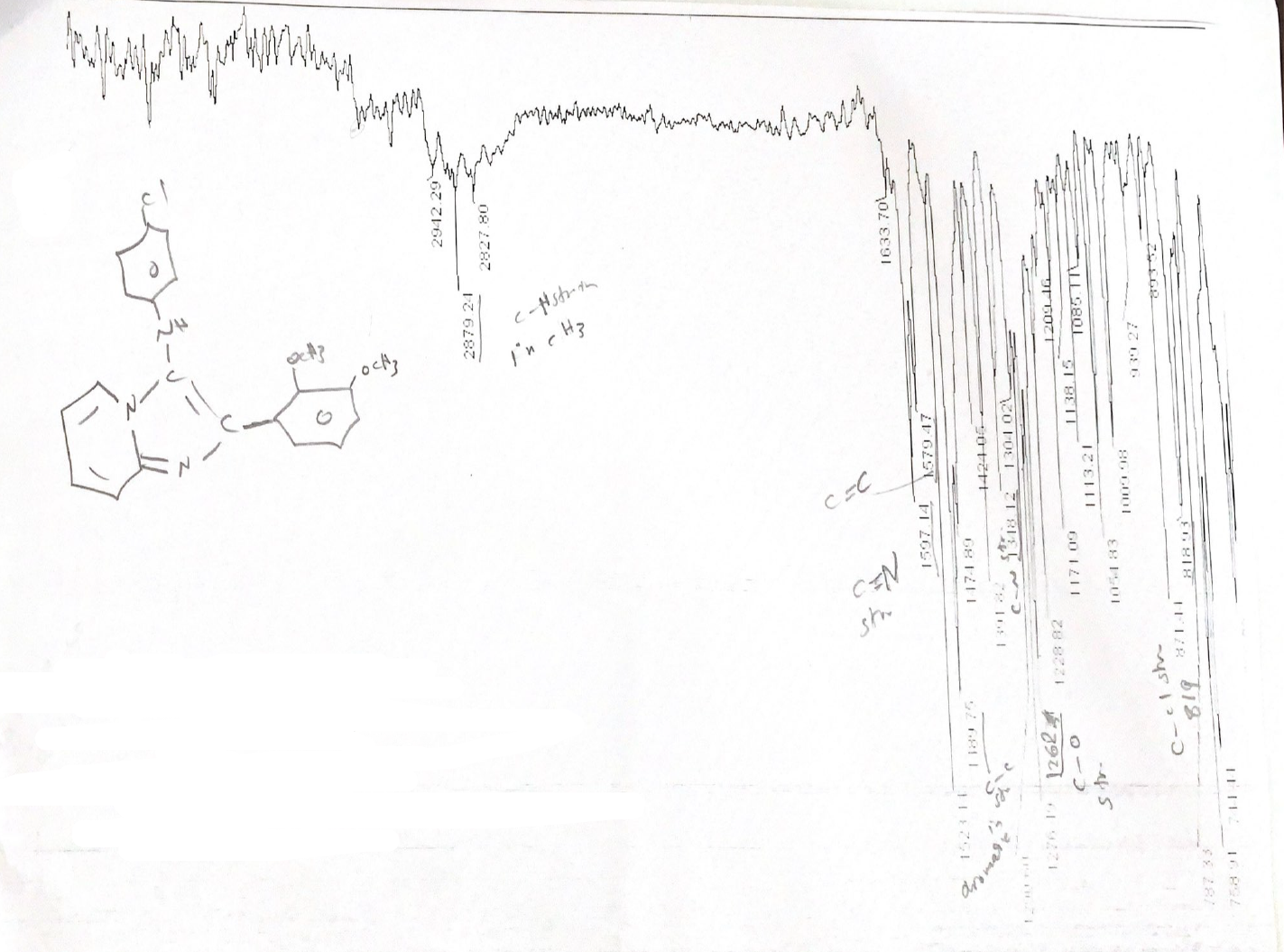
*

**Figure S24.** IR spectrum of compound **15**.

**Figure S25.** ^1^H and ^13^C spectrum of compound **15**.

**Figure S26.** HPLC chromatogram of compound **16**.


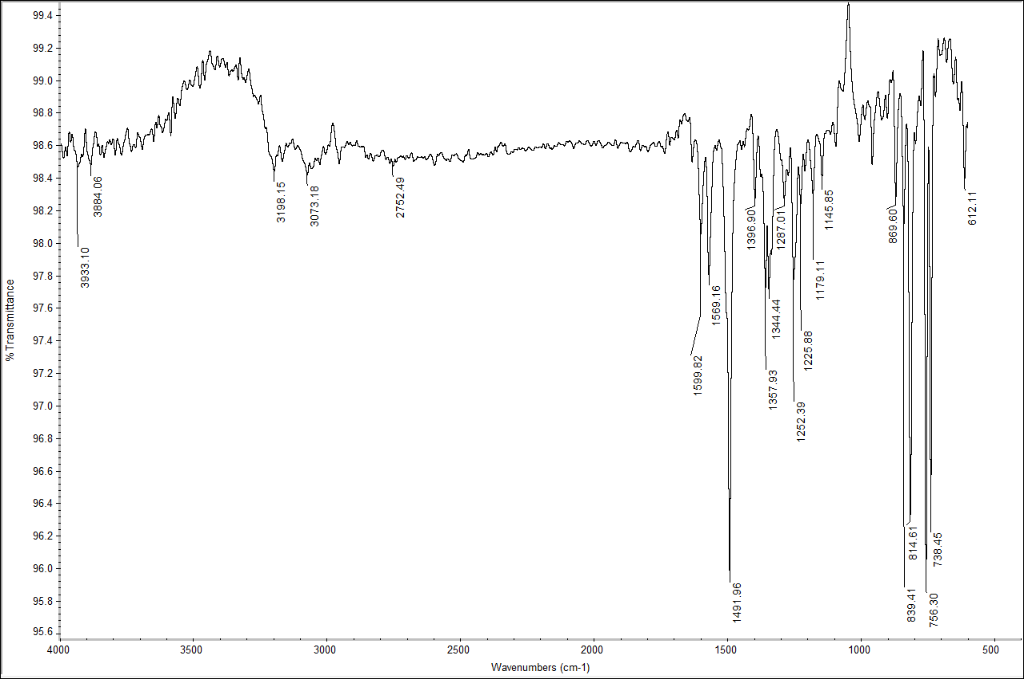


**Figure S27.** IR spectrum of compound **16**.

**Figure S28.** ^1^H and ^13^C spectrum of compound **16**.

**Figure S29.** HPLC chromatogram of compound **17**.


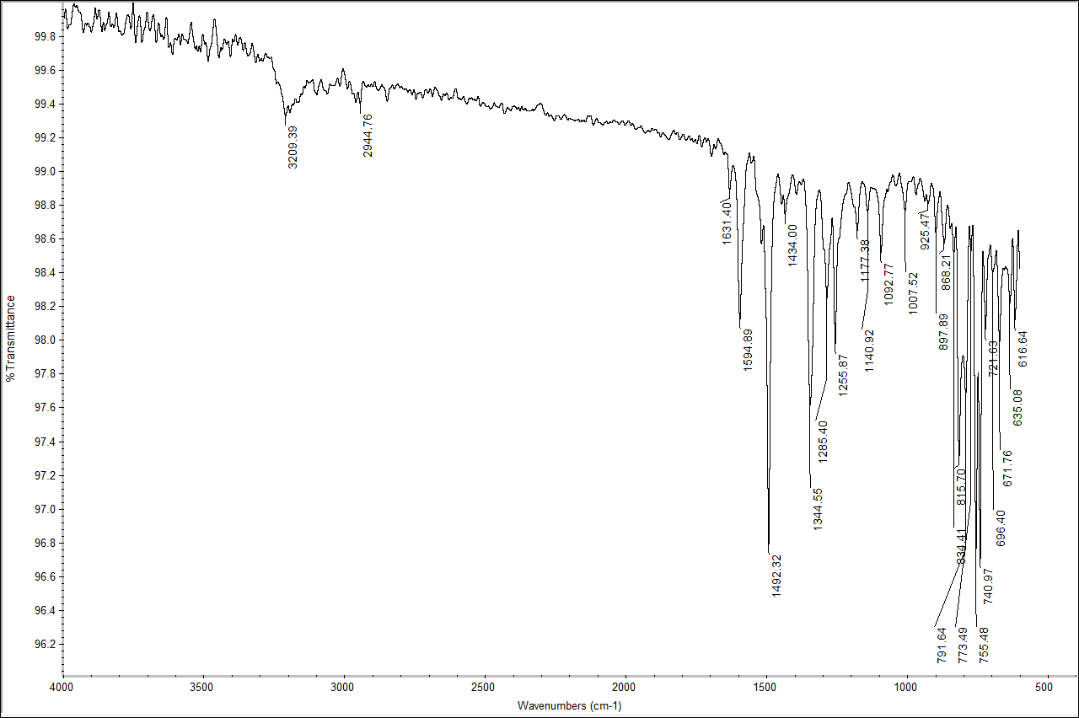


**Figure S30.** IR spectrum of compound **17**.

**Figure S31.** ^1^H and ^13^C spectrum of compound **17**.

**Figure S32.** HPLC chromatogram of compound **18**.


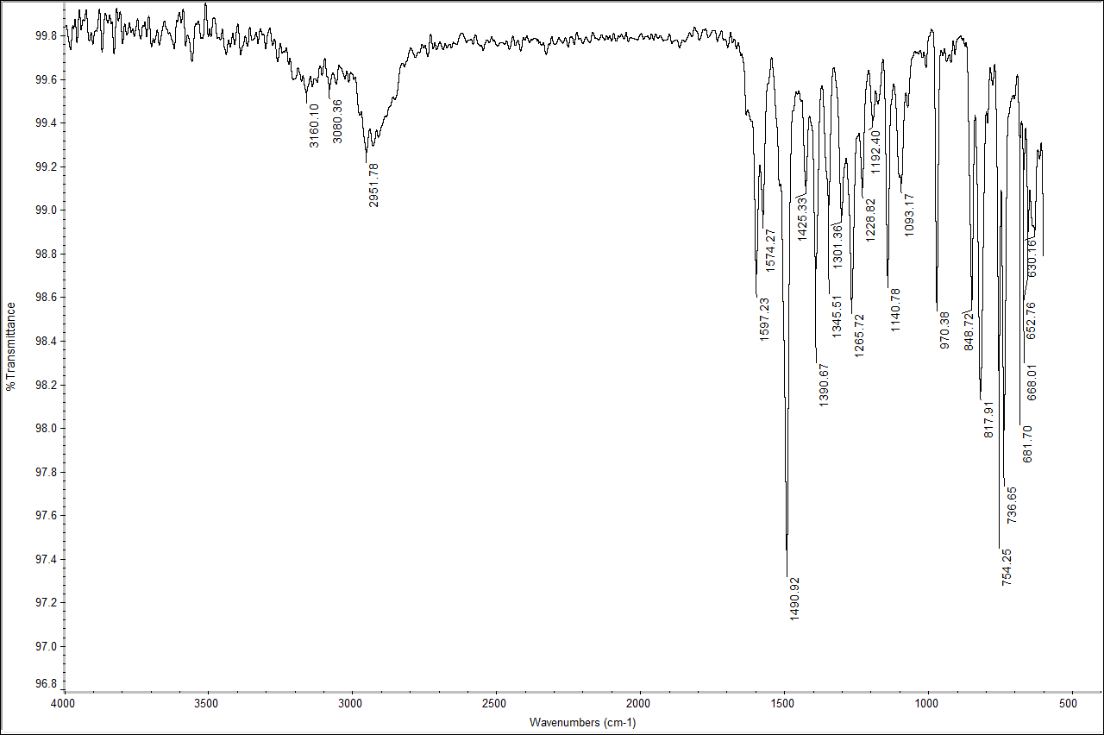


**Figure S33.** IR spectrum of compound **18**.

**Figure S34.** ^1^H and ^13^C spectrum of compound **18**.

**Figure S35.** ^19^F NMR spectrum of compound **18**.

**Figure S36.** HPLC chromatogram of compound **19**.


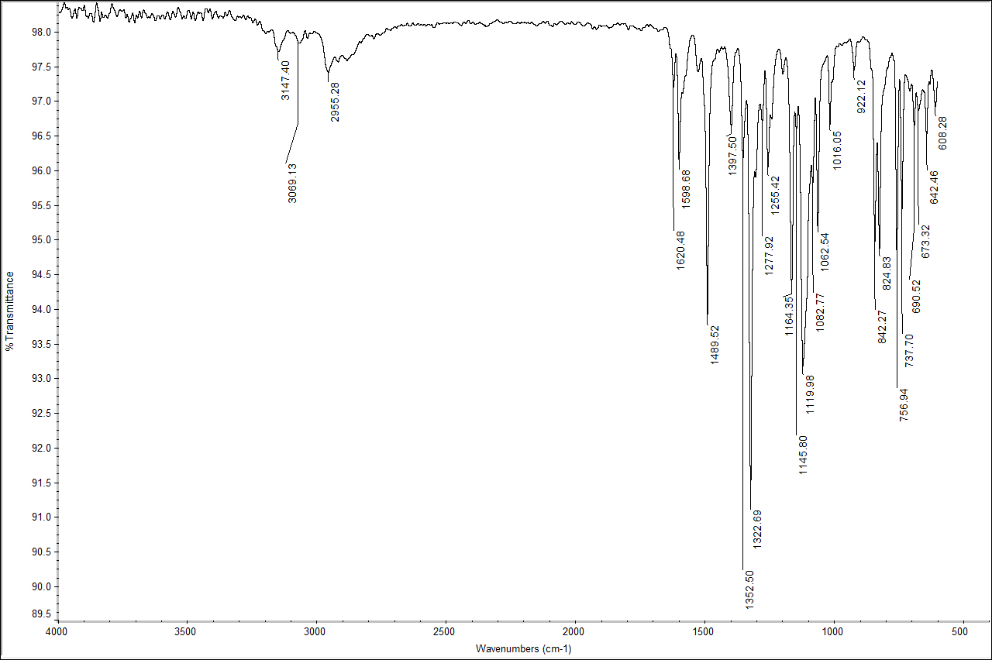


**Figure S37.** IR spectrum of compound **19**.

**Figure S38.** ^1^H and ^13^C spectrum of compound **19**.

**Figure S39.** ^19^F NMR spectrum of compound **19**.
